# Unbiased peptoid combinatorial cell screen identifies plectin protein as a potential biomarker for lung cancer stem cells

**DOI:** 10.1038/s41598-019-51004-3

**Published:** 2019-10-18

**Authors:** Aaron C. Raymond, Boning Gao, Luc Girard, John D. Minna, D. Gomika Udugamasooriya

**Affiliations:** 1 Department of Pharmacological & Pharmaceutical Sciences, University of Houston, 4849 Calhoun Rd, Houston, TX 77204-5037 USA; 2 Department of Cancer Systems Imaging, MD Anderson Cancer Center, 1881 East Road, Houston, TX 77030-4009 USA; 3 Hamon Center for Therapeutic Oncology Research, University of Texas Southwestern Medical Center, 6000 Harry Hines Blvd., Dallas, TX 75390 USA; 4 Simmons Comprehensive Cancer Center, University of Texas Southwestern Medical Center, 6000 Harry Hines Blvd., Dallas, TX 75390 USA; 5 Departments of Pharmacology, University of Texas Southwestern Medical Center, 6000 Harry Hines Blvd., Dallas, TX 75390 USA; 6 Internal Medicine, University of Texas Southwestern Medical Center, 5323 Harry Hines Blvd., Dallas, TX 75390 USA

**Keywords:** Biological techniques, Cancer, Chemical biology, Stem cells, Biomarkers, Oncology, Chemistry

## Abstract

Tumors often contain a small subset of drug-resisting, self-renewing, and highly metastatic cells called tumor initiating cells or cancer stem cells (CSCs). To develop new approaches to detecting and targeting lung cancer CSCs, we applied an “unbiased” peptoid combinatorial cell screen to identify highly specific ligands that bind a CSC subpopulation of non-small cell lung cancer cells (defined by Aldefluor positivity), but not the remaining aldefluor negative cancer cells from the same preclinical model. One of the ‘hit’ peptoids bound to plectin, a structural protein, predominantly expressed intracellularly, but whose localization on the cell surface is linked to tumor invasion and metastasis. Our studies show both genotypic and phenotypic correlations between plectin and lung CSCs, as well as association of high plectin mRNA expression with poor patient survival in lung adenocarcinoma, potentially identifying plectin as a biomarker for lung CSCs.

## Introduction

**Figure 1:**
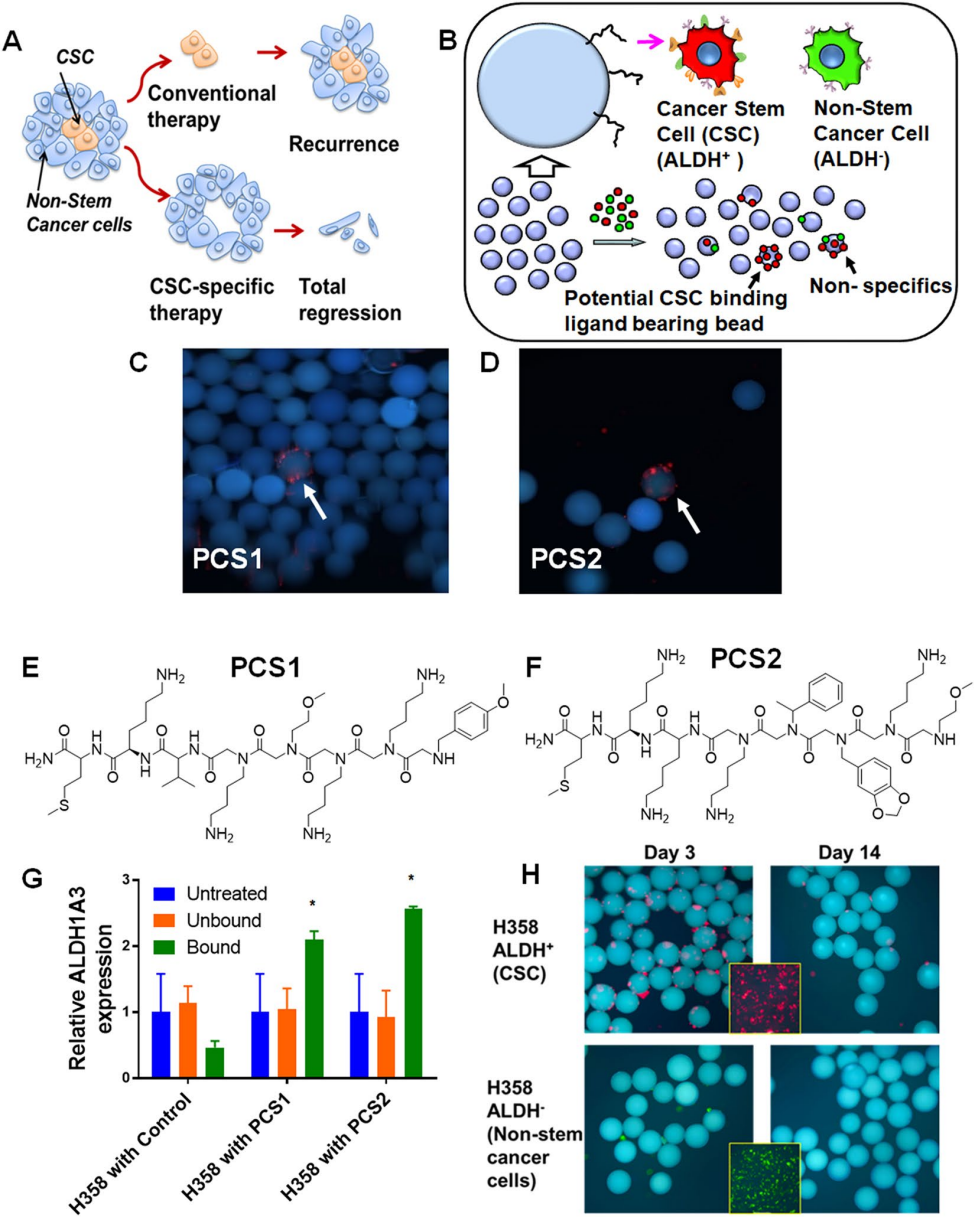
Identification of ALDH^+^ CSC-binding Peptoids. (**A**) Conventional therapies may eliminate the majority of cancer cells, but drug resistant subpopulations such as CSCs may remain intact, which can regenerate the tumor. Therapies directed towards eliminating CSCs could support complete elimination of the tumor. (**B**) Outline of the on-bead two-color (OBTC) Assay. H358 cells were sorted into ALDH^+^ (CSCs: stained red) and ALDH^−^ (non-CSCs: stained green), were mixed 1:1 and exposed to bead (blue) library carrying peptoids on one-bead one-compound format. A bead binding only red cells indicates that the peptoid displayed on that bead recognizes a molecule only (or predominantly) found on CSCs, which is absent (or less abundant) on remaining cancer cells. (**C**,**D**) Beads (blue) bound only by red cells were picked as carrying highly CSC specific ‘hit’ peptoids, PCS1 and PCS2. (**E**,**F**) The structures of ‘hit’ peptoids PCS1 and PCS2, respectively. (**G**) Relative expression of *ALDH1A3* was higher in PCS1 and PCS2 coated magnetic bead bound fractions than unbound or untreated H358. Error bars represent standard deviation between triplicates. *Represents p value < 0.05. (**H**) PCS2 displayed on tentagel beads preferentially recognize ALDH^+^ cells. PCS2-carrying tentagel beads bind to sorted and red Qdot stained ALDH^+^ cells at the day 3 (top row, left panel), but not to green Qdot stained ALDH^−^ (bottom row, left panel). This binding ability of ALDH^+^ cell group was greatly reduced after 2 weeks (top row, right panel). Insert boxes are the red stained ALDH^+^ and green stained ALDH^−^ cells. See also Supplementary Figs. S1–S2, and Table S1.

Despite significant advances in targeting cancer by understanding its “hallmark” molecular mechanisms^1^, most cancer treatments are unable to eliminate small sub-populations of therapy resistant cancer cells, which often have properties of cancer stem cells (CSCs) or tumor initiating cells^2^. These cells can self-renew, divide asymmetrically, and form tumors in isolation^2^. CSCs have been linked to drug resistance, metastasis and tumor relapse^1,3^ and it would be important to discover treatments targeting these sub-populations to provide improved outcomes (Fig. 1A). Developing treatment strategies targeting CSCs remains a challenge, due to the paucity of reliable biomarkers identifying such subpopulations. To circumvent this limitation, we applied our on-bead two-color (OBTC) combinatorial cell screen^4–6^ technology to unbiasedly select peptidomimetic (peptoids: oligo-N-substituted glycines) ligands that specifically target biomarkers on the CSC surface of a preclinical model of non-small cell lung cancer (NSCLC), while not binding to the remaining cancer cells in the same model. Using this approach we identified plectin as a new lung CSC biomarker and potential therapeutic target.

Currently, the most common CSC targeted drug discovery efforts are based on probing developmental pathways that maintain stem cell-like states, such as the Notch, Hedgehog, and Wnt^7^, or to target known biomarkers that are enriched in CSCs such as CD44, ALDH isozymes, and VEGFR^8^. Here, we utilized an ‘unbiased’ selection strategy involving a large peptoid library to identify new lung CSC cell surface biomarkers. Previously reported unbiased cell surface ligand selection methods have used phage display^9^ or synthetic combinatorial screens that subtract background after^10^ or during the initial screens^4,11^. In addition, a preclinical model of breast cancer mesenchymal transformation enriching for CSCs was screened for selective CSC toxicity using a library of ~16,000 chemicals and identified salinomycin as having CSC selective toxicity^12^. We previously reported an OBTC peptoid library screen selection strategy, with cells engineered to express or not express specific biomarkers, allowing us to identify peptoids specifically targeting known cell surface markers such as VEGFR2^4^ and T-cell receptors^5^. We then applied the OBTC technique to distinguish lung cancer cells from normal lung epithelial cells derived from the same patient^6^, and identified lipid-phosphatidylserine as the target of the peptoid-ligand selected from this screen^13^. Here we improved our OBTC technique to unbiasedly screen a large peptoid library for compounds that would bind to a subpopulation of a NSCLC cell line with CSC properties but not to the remainder of the tumor cells from the same cell line, and using these ‘hit’ compounds we identified plectin as a new lung CSC biomarker.

Plectin plays an important role as a bridge between the actin filament and intermediate filament networks^14^ binding to both vimentin^14^ and integrin beta-4^15^. Plectin also plays critical roles in cell-cell signaling and mobility^16,17^. Even though in almost every mammalian cell plectin is housed in the cytosol^18^, a previous study reported plectin as a “mislocalized” cell surface biomarker for pancreatic ductal adenocarcinoma, where it is transported to the cell surface through exosome transport^19^. Plectin “mislocalization” to the cell surface then appears to drive migration and invasion^19^. While these functions are also highly relevant to CSCs, to the best of our knowledge, plectin has not been previously involved as a CSC biomarker. In this study, we show that plectin is highly expressed on the surface of subpopulations of tumor cells within a panel of NSCLC cell lines. These plectin (+) subpopulations are highly clonogenic and enriched for cell migration and other properties of CSCs, and variably correlate with expression of previously described CSC markers such as *ALDH1A3*, *CD44* and *SOX2*.

## Results

### OBTC peptoid library screen identifies highly specific peptoids targeting CSCs present in lung adenocarcinoma line NCI-H358

For our preclinical model, we used NCI-H358 (H358) (p53 null, KRAS mutant, STK11 wild-type), a lung adenocarcinoma cell line consisting of 5–18% of aldehyde dehydrogenase positive (ALDH^+^) cells, a frequently used biomarker for CSCs in cancer cell lines. The subpopulation of H358 ALDH^+^ cells express the *ALDH1A3* isozyme^20^, and are highly tumorigenic compared to the remaining ALDH^−^ cells and thus exhibit CSC/tumor initiating cell like properties^21^. H358 depends on *ALDH1A3* activity, and when *ALDH1A3* is selectively silenced genetically or pharmacologically (inhibiting pSTAT3 or EZH2), the CSC component is lost^20^. Thus, we used a well characterized NSCLC preclinical model to study CSCs. We first isolated ALDH^+^ cells from H358 using the commercially available ALDEFluor assay kit, which is based on the activity of ALDH^20,21^. It is important to note that ALDH is an intracellular protein and our OBTC method is designed to target cell surface molecules, which helps remove bias from our selection method. The ALDH^+^ subpopulation cells were labeled with red Qdots, and the ALDH^−^ subpopulation cells were labeled with green Qdots, mixed 1:1 and equilibrated with 400,000 one-bead one-compound peptoid library (each bead contains a unique peptoid with multiple copies) described previously^6^ (Supplementary Fig. S1). If a bead binds red cells (ALDH^+^) exclusively this indicates that the peptoid on that bead binds to a biomolecule predominantly found on ALDH^+^ cell surface, and not found (or less) on the remaining cancer cells (Fig. 1B). If the targeted marker is expressed on both ALDH^+^ and ALDH^−^ cells, then both red and green labeled cells will bind, and finally, if the bead is covered with a peptoid selective for ALDH^−^ cells, then only green cells would bind (Fig. 1B). Here we identified 2 beads selective for binding red cells (out of 400,000 beads), and the peptoid sequences were identified using Edman sequencing (Supplementary Fig. S1 and Table S1). The specific peptoids were resynthesized for further testing and validation and were named PCS1 and PCS2 (Fig. 1C–F). To validate the binding specificity towards the subpopulation of ALDH^+^ cells, we coated streptavidin-magnetic beads with each of the biotinylated peptoid, mixed with unsorted ALDH^+^ and ALDH^−^ H358 cells, and isolated bound cells. Using RT-qPCR, we found that the expression of *ALDH1A3* gene was higher in both of the peptoid-bound subpopulations compared to unbound, untreated, and controls (Fig. 1G).

### Identified peptoids recognize distinct subsets of lung cancer cells over normal cells

We evaluated the binding potential of peptoids PCS1 and PCS2 on 12 molecularly characterized NSCLC lines and one immortalized normal lung bronchial epithelial cell line (HBEC3KT). We used each of the biotinylated PCS1 and PCS2 (see Supplementary Figs. S3-S4) peptoid-coated magnetic beads to pull-down cells and bound and unbound cells were counted and quantified. None of the peptoids bound to HBEC3KT despite the fact that this cell line exhibits 20–30% ALDH^+^ cells. Peptoids PCS1 and PCS2 bound to distinct subsets of cells within the NSCLC lines (Table 1) and PCS2 displayed a stronger binding to subsets from the majority of NSCLC lines than PCS1. Of interest, despite using ALDH^+^ H358 cells as the criteria for peptoid library screen, binding had no correlation to previously established ALDH activity in the cell line panel^21^. For instance, PCS2 bound to higher percentage of H460 cells, which has only 1% ALDH^+^ cells, and yet does not bind to H1693, which has 38% ALDH^+^ cells. This further confirms that our OBTC selection has no bias towards ALDH expression and activity.

**Table 1:** Relative binding percentage of PCS1 and PCS2 across 13 Lung cell lines.

Relative binding				% of ALDH^+^^*^
Cell line	PCS1	PCS2	Control	
HBEC3KT	−	−	−	20–30%
H2009	−	++++	−	ND
HCC4017	++	+++++	−	ND
H460	++	+++++	−	0.7%
H1975	−	+++++	−	1.2%
H2122	−	+++	−	1.3%
H2073	−	+++++	−	1.4%
H1993	−	+++	−	2.4%
H1299	−	+++++	−	2.9%
H1155	−	−	−	3.0%
H1395	++	+++	−	7.4%
H358	+	+++	−	17.0%
H1693	−	++++	−	38.0%

Relative binding percentage of PCS1 and PCS2 across 12 NSCLC lines and one normal bronchial epithelial line HBEC3KT. The right most column indicates the percentage of cells that are ALDH^+^ in an ALDH ALDEFLUOR assay. PCS1 and PCS2 have distinct binding patterns against 12 NSCLC lines, but do not correlate with previously published ALDH^+^ cell percentages (right most column). PCS1 and PCS2 did not bind to normal bronchial epithelial cells (HBEC3KT). Control compound shows no binding to any cell line. *Sullivan *et al.* Cancer Res. 2010 ND = No Data; PCS1 and PCS2 binding scores are: − = <1%; + = 2–10%; ++ = 11–20%; +++ = 20–30%; ++++ = 31–40%; +++++ = >40%.

### PCS2 recognizes a subpopulation of H358 cells with CSC-like characteristics

To confirm that PCS2 is binding preferentially to ALDH^+^ cells, we exposed PCS2-coated tentagel beads to red stained ALDH^+^ H358 and green stained ALDH^−^ H358 cells, 3 days and 14 days after sorting. PCS2-beads bound significantly to the red cells on the 3^rd^ day and not to green stained cancer cells, as expected (Fig. 1H, first panel). It is known that ALDH^+^ tumor cells will lose ALDH^+^ (CSC) characteristics over time, as they differentiate into non-stem cancer cells^21^. At two weeks, at which point the number of CSCs in the ALDH^+^ sorted population should have decreased through differentiation, red cell binding was decreased (Fig. 1H, second panel).

**Figure 2:**
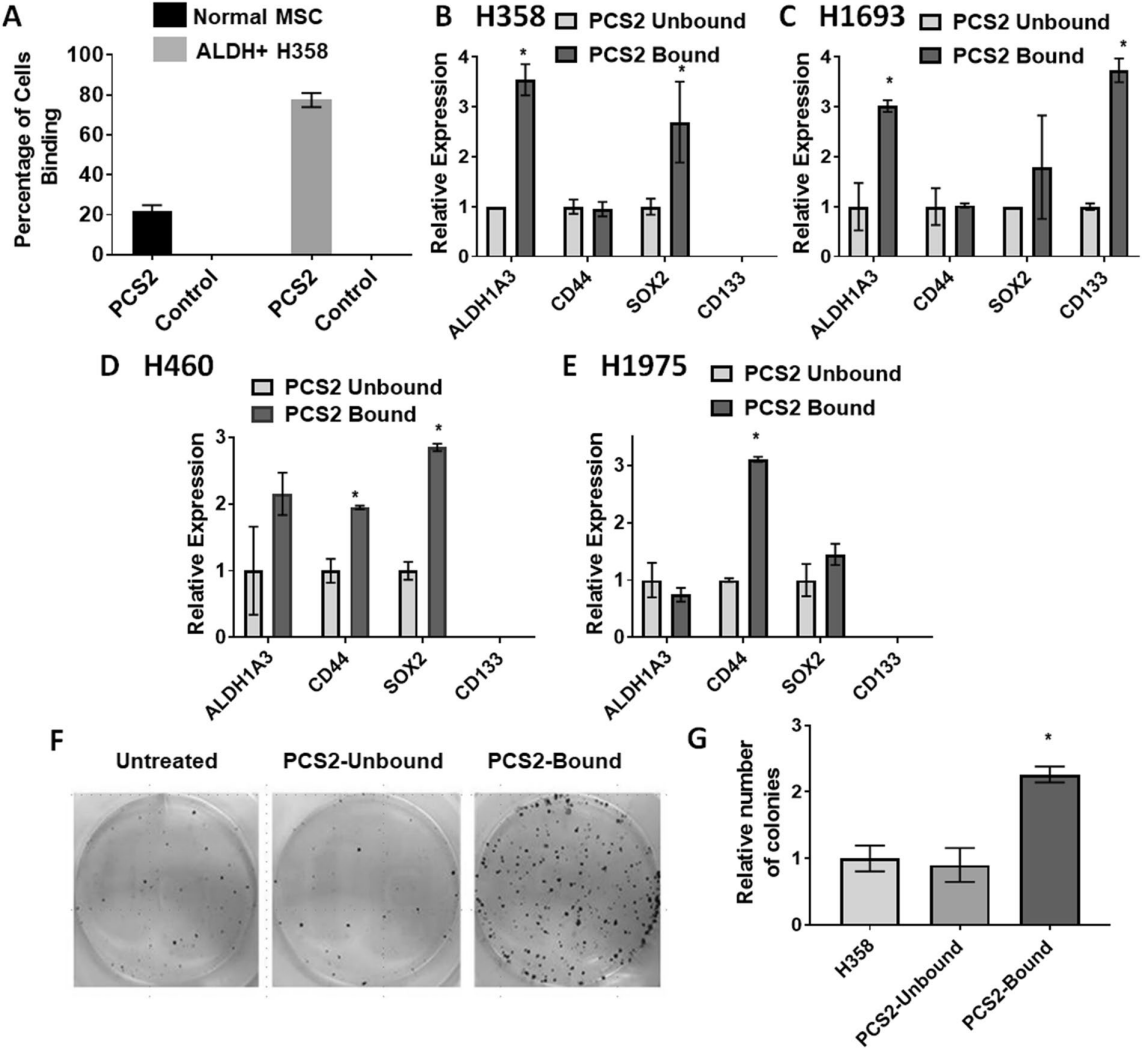
PCS2 binds to a cell fraction with CSC characteristics. (**A**) ALDH^+^ H358 (sorted fraction) and human adipose mesenchymal stem cells (MSC) cells incubated with PSC2 bound beads. ALDH^+^ H358 cells have a higher affinity for PCS2-coated magnetic-beads than MSCs. (**B–E**) RT-qPCR analysis of expression of *ALDH1A3*, *CD44*, *SOX2 and CD133* CSC markers in PSC2 coated magnetic bead-bound and unbound cells. In general, PCS2-bound fractions have higher expression of *ALDH1A3*, *SOX2 and CD133* CSC specific biomarker genes, when detectable, compared to unbound fractions. Cell lines tested were (**B**) H358, (**C**) H1693, (**D**) H460, and (**E**) H1975. H460 and H1975 additionally had significantly higher *CD44* expression on PCS2-bound fractions. (**F**,**G**) Colony formation of H358 cells separated using PCS2-coated magnetic-beads. PCS2 bound cells formed significantly more colonies after 2 weeks of growth than unbound or untreated fractions, indicating PCS2 is pulling down cells with CSC-like characteristics. Error bars represent standard deviation between triplicates. *Represents p value < 0.05.

We focused on peptoid PCS2, due to its higher binding potential across the majority of NSCLC lines tested. Magnetic-bead binding assays showed PCS2 bound to 80% of ALDH^+^ H358 cells compared to 20% of human adipose derived mesenchymal stem cells (MSCs) (Fig. 2A), suggesting the cell surface target of PCS2 has some cancer specificity. We compared RT-qPCR expression in PCS2 bound and unbound NSCLC cells of CSC related biomarkers *ALDH1A3*^20^ *CD133*^21,22^, *CD44*^23^ and *SOX2* [*SRY* (*sex determining region Y*)*-box 2*]^7^ using H358, H1693, H460, and H1975 NSCLC lines (Fig. 2B–E). *ALDH1A3* and *SOX2* expression was higher in PCS2-bead-bound cells for H358, H1693, and H460, while *CD44* expression was substantially higher in PCS2-bead-bound H460 and H1975 cells. *CD133* expression was higher only in PCS2-bead-bound H1693 cells and undetectable in all three remaining cell lines. It is known that the expression of two lung CSC biomarkers ALDH and CD133 do not correlate^21,24,25^, and we did not detect CD133 expression in either the PCS2 bound or the unbound fractions of H358, H460, and H1975 NSCLCs (Fig. 2B–E). We next evaluated the colony formation potential, an *in vitro* characteristic trait of CSCs that is a well-established model for tumorigenic potential. Briefly, once cells are seeded at very low concentration, they spread over the plate and lose cell-cell communications that is essential for cell survival. But, CSCs can survive alone and they start forming colonies in isolation. We found that PCS2-bound H358 cells established more colonies than unbound and unsorted H358 cells (Fig. 2F,G), indicating PCS2 isolated CSCs. All these studies are consistent with the proposal that PCS2 binding identified a subpopulation of H358 cells with CSC-like characteristics.

### Plectin is the putative target of PCS2

**Figure 3:**
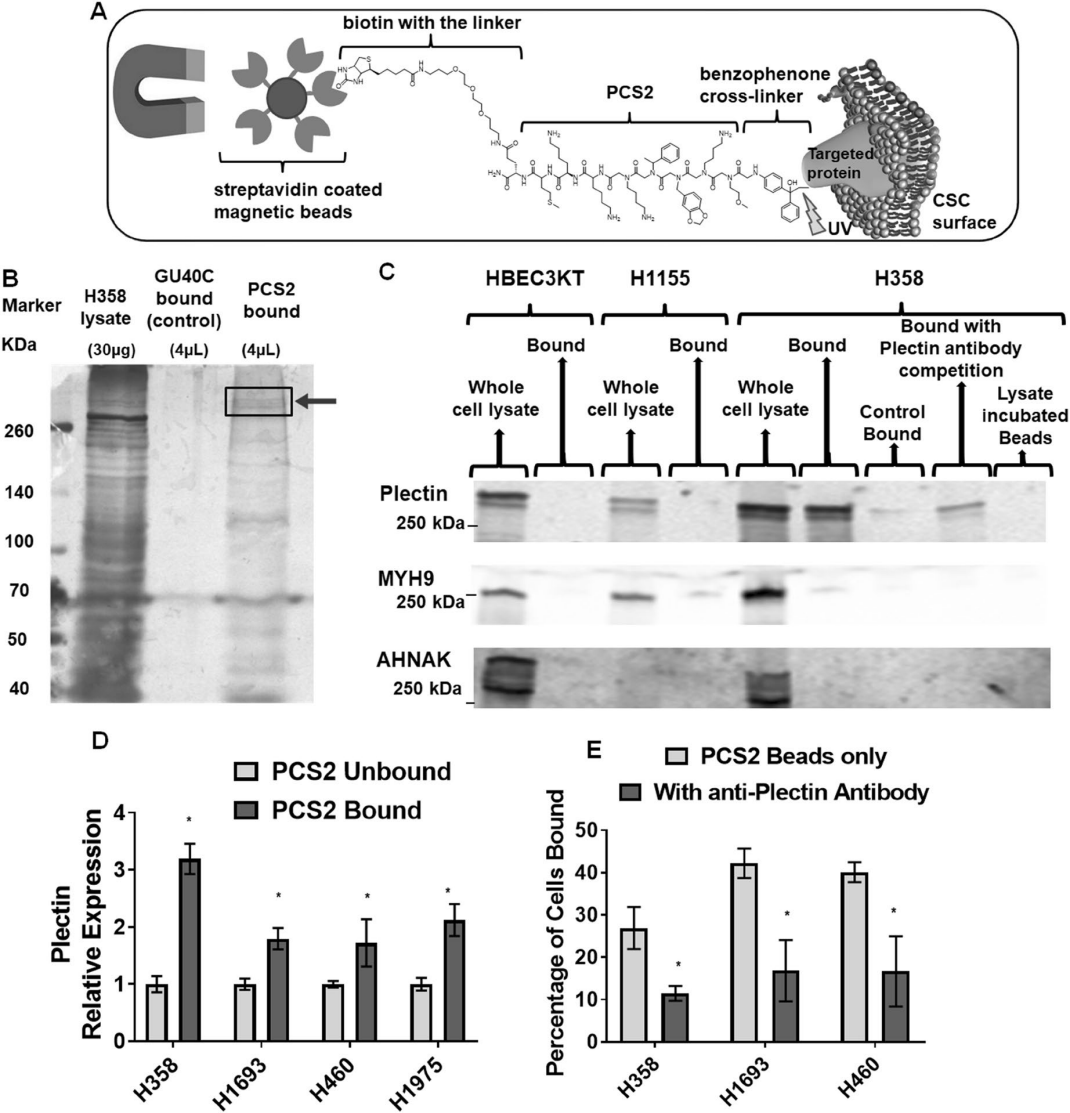
Plectin is the putative target of PCS2. (**A**) Outline of the benzophenone-PCS2-coated magnetic bead pull-down approach used for proteomics based target identification (see Supplementary Fig. S5 for peptoid characterization). (**B**) Target pull-down from H358 cell surface shows unique protein bands at ~500 kDa, which was not found in the leftover cell lysate and control pull-down fraction. The standard proteomics analysis indicated that plectin protein was the potential hit in this region. The gel is cropped, and full length blots are presented in Supplemental Fig. S8. Note: This assay was repeated 3 times, and in all cases plectin protein was pulled down as most likely target (See Supplementary Proteomics Dataset S1, 2, 3 and 5), while MYH9, or AHNAK proteins also could have been possible targets (See Supplementary Proteomics Dataset S3 and 4). (**C**) Western blot of the PCS2-target pulldown in HBEC3KT, H1155, and H358 cells, immunostained with Plectin, MYH9, and AHNAK antibodies. Only plectin was detected in the PCS2-bound fraction of H358 while no PCS2 binding occurred in HBEC3KT or H1155, indicating the target of PCS2 is plectin. The anti-plectin antibody incubated during H358 cell binding to benzophenone-PCS2-coated magnetic beads reduced the intensity of the plectin band, further confirming plectin as the target. In addition, benzophenone-PCS2-coated magnetic beads were incubated with H358 lysate to see whether cytosolic proteins would nonspecifically attach to the bead surface, but no bands were observed, indicating this has not occurred. MYH9 and AHNAK antibodies were used as non-specific controls, as they were identified, non-reproducibly, as potential targets in a single pull-down replicate (Supplementary Proteomic Dataset S3, S4). But none of the bound fractions display significant bands. The blots are cropped, combined from multiple gels (that were repeated at least 3 times), and full length blots are presented in Supplemental Fig. S9. (**D**) PCS2-coated magnetic-beads bound fractions of H358, H1693, H460, and H1975 cells have higher expression of plectin (*PLEC*) compared to unbound and unsorted fractions. (**E**) The inclusion of anti-plectin antibody significantly decreases the percentage of cells binding to PCS2-coated magnetic-beads on those H358, H1693, and H460 cells. Error bars represent standard deviation between triplicates. *Represents p value < 0.05.

To identify the cellular target of PCS2, we performed a pull-down assay using streptavidin-magnetic beads coated with biotinylated-PCS2-benzophenone (see Supplementary Fig. S5). When PCS2 binds to its corresponding targeted protein, the photo-affinity probe benzophenone can be activated with wavelength-specific light (UV), which generates a highly reactive radical that covalently cross-links with the target protein (Fig. 3A)^26^. Previously published VEGFR2 targeted biotinylated-GU40C-benzophenone peptoid was used as the non-binding control ligand^4^. We allowed biotinylated-PCS2-benzophenone and biotinylated-GU40C-benzophenone bind to H358 cells, separated the bound cells using magnetic beads, cross-linked using UV, lysed cells, separated proteins by electrophoresis and visualized these proteins by silver staining. The unique band at around 500 kDA in the PCS2-bound sample, and not present in the control-bound sample nor enriched in the leftover cell lysate (not bound to PCS2) (Fig. 3B), was analyzed by standard proteomics technique. Out of the candidates identified in this region, only plectin was reproducibly detected in multiple experiments (Supplementary Proteomics Dataset S1, 2, 3 and 5). Since this was the only reproducible target, we chose to focus on plectin as the potential hit.

To verify plectin as the target, we isolated the PCS2 bound protein by target-pull-down (described in Fig. 3A), ran the isolate on a gel again, but this time performed a Western Blot using the antibodies for three proteins; plectin and 2 of the non-reproducible, but possible pull-down targets, Myosin 9 (MYH9), and Neuroblast differentiation-associated protein (AHNAK). On H358, plectin was found in the PCS2-bound sample and whole cell lysate, but not in the control-GU40C-benzophenone bound sample (Fig. 3C), identifying plectin as the most likely target. We note, both AHNAK and myosin have known protein-protein interactions with plectin so it is possible their detection in these pull down assays resulted from their interactions with plectin itself^27^. In addition, when this magnetic-bead coated PCS2 binding assay was performed in the presence of the commercially available plectin specific antibody, the PCS2 bead binding and plectin detected by Western blot was dramatically reduced (Fig. 3C). As further confirmation, no plectin bands were detected when NSCLC line H1155, or normal HBEC3KT, neither of which bind PCS2 (Table 1), were used (Fig. 3C). Exposure of magnetic beads to H358 whole cell lysate also did not produce a plectin band, indicating plectin signal was not due to nonspecific adherences of plectin to these beads. No MYH9 and AHNK bands were detected on PCS2-bound samples from any of the 3 cell lines. We then tested plectin expression on PCS2-bound cells from H358, H1693, H460, and, H1975 cells by RT-qPCR. *PLEC* expression was significantly higher in PCS2-bound cells than unbound cells in all four lines (Fig. 3D). This result suggests that the plectin protein not only mislocalize to the cell surface of these PCS2-bound cells but is also expressed at a higher level in these cells. We also found that an anti-plectin antibody blocked the binding of NSCLCs (H358, H1693, and H460) to the PCS2 coated beads (Fig. 3E). From all these observations, we conclude that plectin is the cell-surface marker that PCS2 is targeting.

### Plectin is found on H358 cell surface, both PCS2 and anti-plectin antibody bind and pulled down cells with CSC signatures

**Figure 4:**
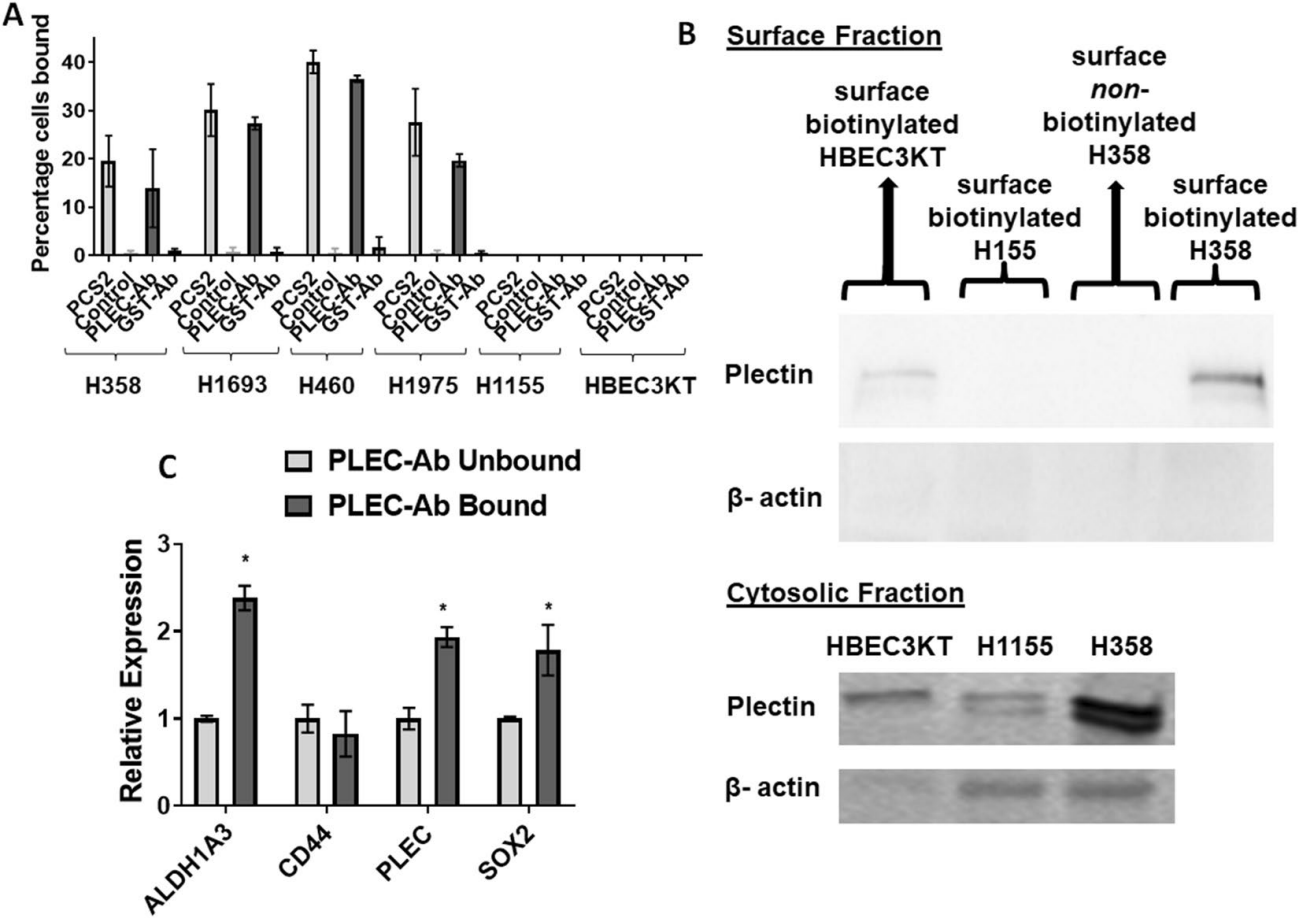
Plectin is found on H358 cell surface and plectin antibody binding identified a subpopulation of H358 cells with CSC-like characteristics in a similar manner to PCS2. (**A**) Plectin antibody-coated magnetic-beads bind to a similar percentage of cells when compared to PCS2-coated magnetic-bead binding in 4 lung cancer lines (H358, H1693, H460, H1975) and had no detectable binding on PCS2-non-binding lung cancer line H1155 and normal HBEC3KT. In comparison, GST antibody-coated and non-binding control compound-coated beads do not bind to any of the cell lines tested. (**B**) The Pierce Cell Surface Protein Isolation Kit (Thermo Fisher) biotinylated only outer cell surface proteins, isolated and separated those from cytosolic proteins. The plectin protein is significantly detected in the cell surface fraction of H358 cells, and not significantly in HBEC3KT, H1155, or H358 without the initial cell surface biotinylation component, while plectin is detected in all 3 lines in the cytosolic fraction. β-actin is used as a cytosolic-specific marker for comparison, which did not appear on the cell surface fraction indicating the purity of the cell surface fraction. The blots are cropped, combined from multiple gels (that were repeated at least 3 times), and full length blots are presented in Supplemental Fig. S10. (**C**) Plectin antibody-coated magnetic-bead bound fraction from H358 have higher expression of *PLEC*, *ALDH1A3 and SOX2* compared to the unbound fraction. Error bars represent standard deviation between duplicates. *Represents p value < 0.05.

First, to determine if the results we are seeing for PCS2 are due to plectin-specific pulldown or other off-target or non-specific effects, we evaluated if a group of cells sorted for cell surface plectin expression through a PCS2-independent approach had similar characteristics. We used the same membrane impermeable magnetic-bead pull-down assay on four PCS2 binding (H358, H1693, H460, and H1975) and two PCS2-non binding lines (H1155, and HBEC3KT) and coated the beads with: (I) biotinylated-PCS2, and (II) an available biotinylated anti-plectin antibody to separately pull-down cells. For each of the four PCS2 binding lines tested, the percentage of cells bound to plectin-antibody coated beads was similar to that of the PCS2-coated beads and the PCS2 binding negative H1155 and HBEC3KT cells also did not bind to anti-plectin antibody coated beads (Fig. 4A). By contrast, the anti-GST antibody and non-binding control compound coated magnetic beads did not pull down any of the cells.

If PCS2 is interacting with plectin, then we would anticipate plectin to be on the cell surface. To show that plectin is indeed expressed on the tumor cell surface, we used Pierce Cell Surface Protein Isolation Kit (Thermo Fisher). This is a commercially available kit specific for detecting cell surface proteins, which identifies and isolates proteins expressed only on the outer cell surface through biotinylation, and not inner cell membrane bound proteins or cytosolic proteins. This kit was a critical necessity for answering this question, since standard cell membrane fractionation methods detect any membrane bound proteins and plectin is typically found on the inner cell membrane under normal conditions. We then performed western blotting using those cell surface and cytosolic protein fractions on H358 and two PCS2-non binding lines, HBEC3KT and H1155. Plectin was significantly detected in the H358 cell surface fraction, but not in H1155 and only trace amounts in the HBEC3KT surface fractions (Fig. 4B). As expected, all 3 cell lines had plectin expression in cytosolic fractions (Fig. 4B). The cytosolic marker, β-actin was readily detected in the cytosol, but not found on cell surface fractions, indicating the purity of these fractions (Fig. 4B).

To further validate that cell surface plectin identifies a potential CSC marker, we analyzed the mRNA levels of the CSC biomarkers previously used (*ALDH1A3*, *CD44*, *SOX2*) on plectin-antibody-bound H358 cell fraction by RT-qPCR, along with plectin (*PLEC*). The expression levels of *ALDH1A3*, *PLEC*, *and SOX2* of the CSC markers were higher in plectin-antibody-bound cells, than in unbound cells (Fig. 4C), indicating the cell subpopulation pulled down from H358 carries those CSC signatures. Also, it is important to note that these data are highly comparable to the data of PCS2-bound fraction of H358 described in Fig. 2B, indicating both PCS2 and plectin-antibody have pulled down the same cell fraction. All these studies indicate that plectin is the protein being detected by the PCS2 peptoid, and the expression of plectin is highly enriched on the H358 cell surface.

### Plectin knockdown decreases CSC characteristics both at genotypic and phenotypic levels

**Figure 5:**
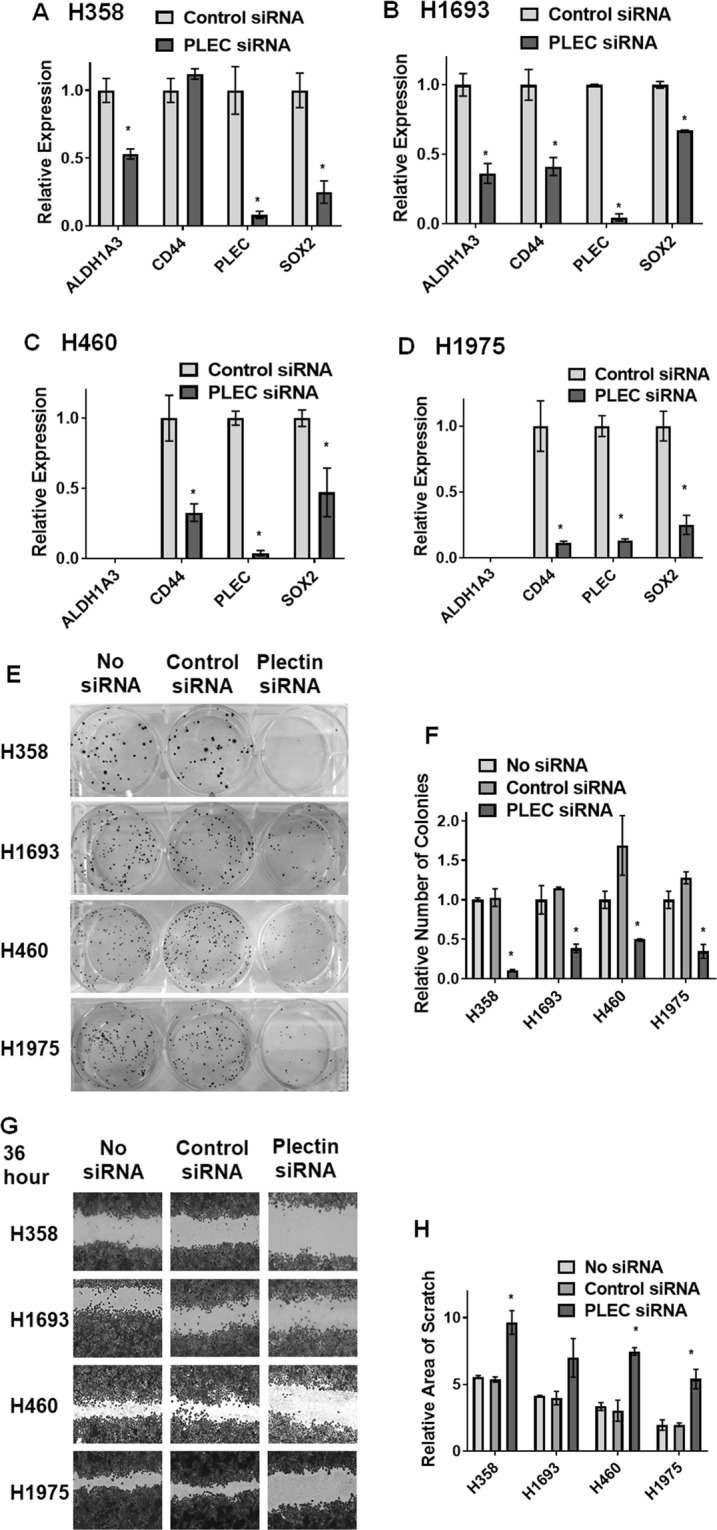
Plectin knockdown decreases CSC characteristics both at genotypic and phenotypic levels. (**A–D**) Plectin siRNA knockdown cultures from four cell lines: (**A**) H358, (**B**) H1693, (**C**) H460, and (**D**) H1975, have decreased expression of *PLEC*, *ALDH1A3 and SOX2*, where detectable, while H1693, H460, and H1975 also show decrease in *CD44*. Error bars represent standard deviation between triplicates. (**E**,**F**) Knockdown of plectin decreases colony formation potential in H358, H1693, H460,and H1975 cells. (**E**) Visualization of colony formation. (**F**) Quantification of colony count. (**G,H**) Knockdown of plectin decreases the mobility and scratch healing in H358, H1693, H460, and H1975 cell lines, incubated for 60 hours with siRNA (36 hours before scratch and 24 hours after). Error bars represent standard deviation between duplicates. *Represents p value < 0.05.

Since previous reports have shown cells with plectin knockdown are sufficiently stable for further assays^19^, we first evaluated if plectin knockdown would have an effect on the gene expression of CSC markers. We performed plectin siRNA knockdown experiment and analyzed the mRNA levels of the CSC markers previously used (*ALDH1A3*, *CD44*, *SOX2*) and *PLEC* in H358 (Fig. 5A), H1693 (Fig. 5B), H460 (Fig. 5C), and H1975 (Fig. 5D). Upon plectin knockdown, expression of *PLEC and SOX2* genes were decreased in all 4 cell lines, while *CD44* decreased in H1693, H460, and H1975, and *ALDH1A3* expression decreased in H358 and H1963.

To identify if plectin knockdown had an effect on the stem cell-related colony formation phenotype,we performed the clonogenicity assay on 4 PCS2-binding cell lines, H358, H1693, H460, and H1975. In all cases, plectin knock down led to reduction of colony formation compared to control siRNA or untreated cells (Fig. 5E,F).

Since plectin expression has previously been shown to be associated with migration and invasion in other cancer types^28–31^, and those phenotypes are associated with CSCs, we performed a “scratch” wound healing/mobility assay under siRNA knockdown conditions in the same 4 cell lines that exhibited strong PCS2 binding potential (H358, H1693, H460, and H1975). In each cell line both untreated and control siRNA treated conditions had a decreased scratch width, after 36 hours siRNA exposure pre-scratch and 24 hours post-scratch, compared to the plectin siRNA knockdown condition, indicating that plectin knockdown reduced the mobility and migration potential of these cells (Fig. 5G,H). This decrease in wound repair was further highlighted when pre-scratch siRNA exposure was increased to 48 hours (Supplementary Fig. S6). However, with long incubation period the pectin knockdown was beginning to have an effect on the health and stability of the monolayer, suggesting plectin is important for either sustained culture health or adherence potential in these lines. The loss of monolayer stability was best represented when the cell cultures were exposed to siRNA for 96 hours without any scratch being made (Supplementary Fig. S6). Overall, these findings indicate plectin can play a role in clonogenicity and migration of NSCLC cells, which are both hallmark characteristics of CSCs.

### Plectin expression correlates with poor patient survival and plectin isoforms 1a and 1f are highly expressed in NSCLC lung cancer

**Figure 6:**
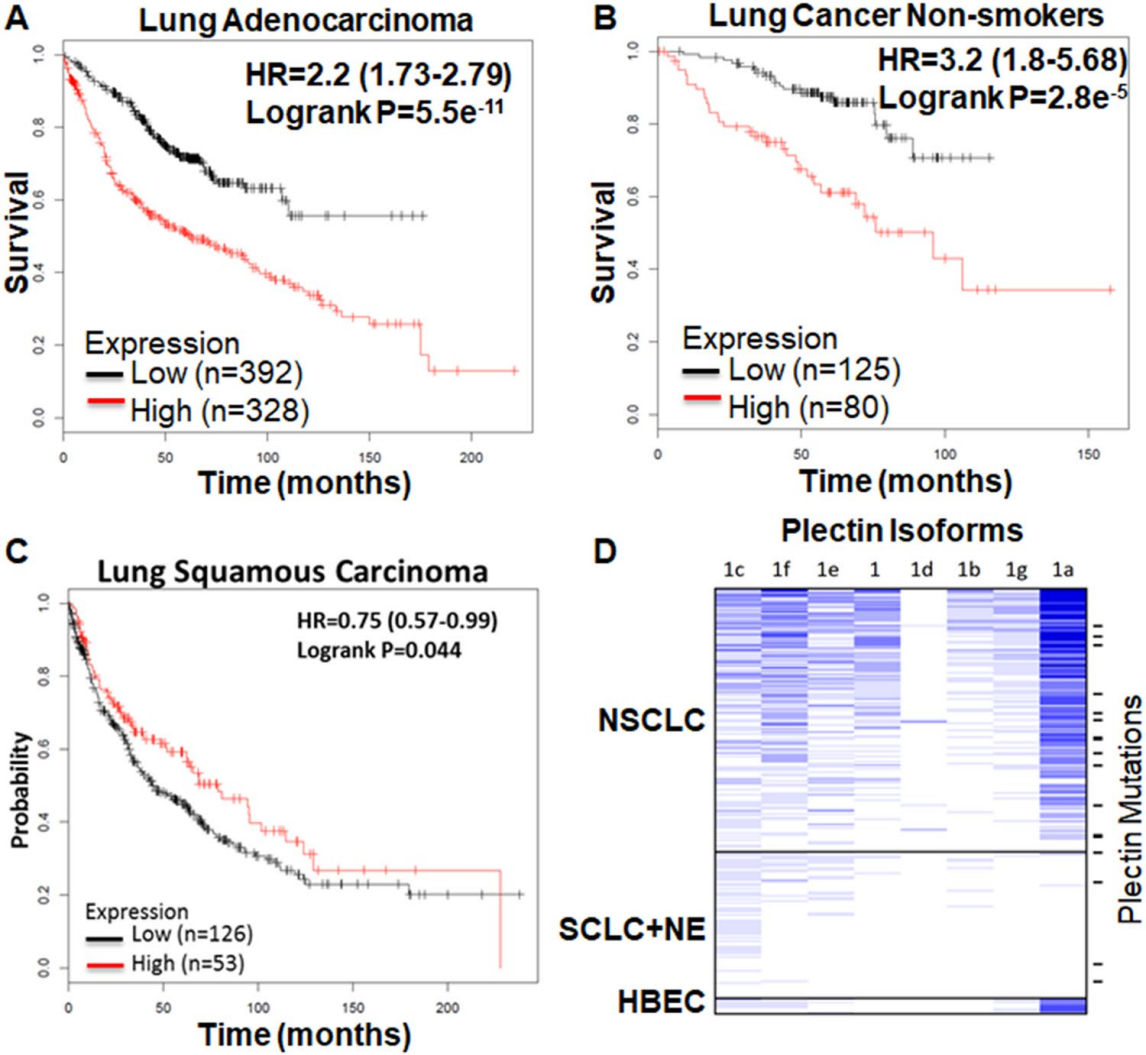
Higher level expression of plectin is associated with poor patient survival in lung adenocarcinoma patients and non-smokers, while opposite observation is on lung squamous carcinoma, and plectin isoforms 1a and 1f are highly expressed in NSCLC patients. (**A–C**) Kaplan–Meier survival plot of NSCLC patients^32^. (**A**) Lung adenocarcinoma, (**B**) non-smokers in NSCLC (adenocarcinoma and squamous cell carcinoma), (**C**) squamous cell carcinoma. Red and black bar indicate the high and low expression of plectin respectively. HR: hazard ratio. (**D**) RNAseq expression of plectin isoforms (1, 1a-1g) in NSCLC (n = 151) and SCLC+ Large Cell Neuroendocrine (NE, n = 84) cell lines. Plectin mutations were found in 14 NSCLC and 4 SCLC/NE cell lines (short bars at right).

We examined the association of patient survival with plectin RNA expression using kmplot (www.kmplot.com/lung)^32^. This database consists of 10 independent data sets of 1926 lung adenocarcinoma and squamous carcinoma patients. Higher expression of plectin is strongly associated with poor survival in patients with adenocarcinoma (HR = 2.2, P = 5.5e^−^^11^, Fig. 6A) and in never smoking lung cancer patients (HR = 3.2, P = 2.8e^−^^5^, Fig. 6B). As an opposing observation, the high plectin expression level is associated with better survival in squamous carcinoma patients (Fig. 6C) although the difference is very small (HR = 0.75, P = 0.044). More detailed studies in lung cancer patients with different histology, gender and smoking status showed that the high plectin expression is associated with poor survival with the only exception of patient with squamous carcinoma (Supplementary Fig. S7). In summary, most of the detailed studies showed high plectin expression is associated with poor patient survival. Results of this analysis which is based on clinical studies further strengthen the notion that plectin plays important roles in tumorigenesis.

There are at least 9 different isoforms of plectin in human. Plectin proteins encoded by isoforms 1a and 1f have been shown to be located on cancer cell surface^19^. We examined the expression of different plectin isoforms in NSCLC lines. Plectin isoform 1a is the highest expressed isoform in NSCLC cell lines, followed by isoform 1f (Fig. 6D). Interestingly, neither isoform is expressed in SCLC cell lines, suggesting that plectin may be a CSC biomarker specific for NSCLC. At least 14 NSCLC cell lines (8%) and 4 SCLC/NE cell lines (6%) have plectin mutation, similarly to TCGA NSCLC (9%). The effect of these mutations is unknown at this point.

## Discussion

In the current study, we have used our OBTC approach to screen a large peptoid library and identified the PCS2 peptoid that specifically bound the ALDH^+^ subset of H358 NSCLC cells, which previously has been shown to be enriched in CSCs^21^. We were able to demonstrate that PCS2 binds to plectin, which normally is expressed intracellularly, but in cancer cells can be expressed on the cell surface. Subpopulations of several NSCLC lines that express plectin and that can be isolated with our PCS2 ligand or an anti-plectin antibody, express elevated levels of stem cell markers such as *ALDH1A3*, *SOX2* and *CD44*. It is important to point out that these biomarkers are not always correlating due to the fact that tumor biomarker expressions are highly heterogeneous, which leads to the existence of various subpopulations within each cancer type^21,33^. The best example in this context is the poor correlation between two lung cancer specific CSC biomarkers: ALDH and CD133^20,21^. Cell surface plectin expressing NSCLC subpopulations isolated by PCS2 binding have increased clonogenicity and migration/invasion characteristics compared to plectin^−^ (plectin absent on the cell surface) NSCLC subpopulations from the same tumor line. Furthermore, knockdown of plectin in NSCLCs results in cells with lower expression of CSC markers, and reduced clonogenicity and migration/invasion. Together these observations indicate that not only does the expression of cell surface plectin identifies subpopulations of NSCLC cells with many properties of CSCs, but is also functionally important for these properties and potentially represents a new therapeutic target.

Plectin is a large protein which is ubiquitously expressed in most mammalian cells. It has a number of isoforms with distinct N-terminal domains^34,35^ that have a similar overall size of ~500 kDa and a structure of two globular domains with a large coiled coil of alpha helices (Rod domain) in between^35–37^. The primary role of plectin is to function as a structural linker between cellular membranes and cytoskeletal components, including the actin microfilaments, microtubules and intermediate filaments^14,38,39^. Through these interactions, plectin plays an important role in cell-cell interactions, cell-extracellular matrix interactions, cell migration, cellular and tissue integrity and plasticity. Loss or mutations in plectin are associated with tissue degeneration and a loss of cellular structural stability, and in mice, plectin knockout is lethal 3 days after birth^40^. Importantly, multiple studies have shown plectin expression to be associated with cancer progression and metastasis^19,29–31,41^. In this regard, higher tumor plectin mRNA expression is strongly associated with poor overall survival in lung adenocarcinoma and in non-smoking patient but not in lung squamous cancer (Fig. 6A–C). To date plectin has not been shown to be co-expressed with known CSC biomarker genes (*ALDH1A1, CD44 and SOX2*), or its expression correlates with CSC phenotypes (clonogenecity and wound healing), and in this study we show that plectin indeed has both genotypic and phenotypic correlations with CSCs.

Recently it was shown that plectin, as part of a plectin-EPLIN (epithelial protein lost in neoplasm)-microtubule complex (in KRASV12 transformed cells), plays an important role in the apical extrusion of KRAS V12 transformed cells from normal epithelial cells and thus activation of the plectin complex could enhance eradication of newly emerging transformed cells from the epithelium^42^, indicating plectin could be a target for both early cancer prevention as well as CSCs in established cancers. There are at least 12 known plectin isoforms in humans, and isoforms 1a and 1f have been found on cancer cell surfaces in pancreatic cancer^19^. Interestingly, our genome-wide RNaseq data also found that plectin isoforms 1a and 1f are highly expressed in NSCLC cell lines, but not in SCLC lines (Fig. 6D).

While the expected localization of plectin is cytoplasmic, cell surface expression in pancreatic cancer had been reported (via exosomes secreted from cancer cells), playing a role in growth of pancreatic cancer xenografts in immunodeficient mice^19^. We hypothesize that such a ‘mislocalization’ of plectin onto cell surface had occurred in NSCLC as well, and that was captured by our PCS2 peptoid in the OBTC screen. Observations in other cancers are consistent with plectin playing a role in CSCs such as promoting migration and invasion in head and neck squamous cell carcinoma^31^. Also, plectin expression correlates with the interacting intermediate filament vimentin to metastatic potential in androgen-independent prostate adenocarcinoma^41^. It is known that CSCs and normal stem cells are both enriched with ALDH^+^ cells^43^. We have previously shown that HBEC3KT, which is enriched with ALDH cells, has the characteristics of normal lung stem cells^44^. In the current study we found that PCS2 bound to ALDH^+^ cells in H358, which has the membrane localized plectin but not to HBEC3KT cells, which lacks the membrane localized plectin. Thus the result is consistent with the notion that the ‘mislocalization’ of plectin onto cell surface is one of the characteristics of CSCs.

In addition to the studies of plectin functions in lung CSCs, we analyzed the expression of plectin in clinical studies and found the high expression of plectin is associated with poor survival of lung adenocarcinoma patients. The association is highly significant with the hazard ratio of 2.2 and p value of 5.5e^−11^. The result of this analysis further strengthen the notion that plectin plays important roles in tumorigenesis. It is interesting that the decrease of plectin associated lung cancer survival is more significant in non-smokers (HR = 3.2, p = 2.8e^−5^, Fig. 6 and Supplementary Fig. S7) than that in smokers (HR = 1.9, p = 4.8e^−4^, Supplementary Fig. S7). The risk factors that cause lung cancer in non-smokers are complex and being actively investigated. Studying the function of plectin may give important insight in the study of non-smoking related lung cancer.

We applied our OBTC assay in an unbiased fashion, designed to identify ligands that bind to biomolecules found on the surface of H358 NSCLC cell subpopulations greatly enriched for ALDH^+^ cells, and importantly counter selected by not being expressed in the ALDH^−^ population in the same tumor line. Even though we isolated plectin binding PCS2 peptoid targeting ALDH^+^ tumor cells, we subsequently found that the binding of PCS2 was independent of ALDH expression. Thus, our study illustrates the power of the OBTC peptoid library screening technology to “unbiasedly” identify biomarkers in defined subpopulations of cancer cells, particularly when the remainder of the tumor cells from the same tumor can be used as real-time controls in the single step screening procedure. The other unbiased approaches such as phage display needs multiple rounds to complete the screen on control cells and is limited to peptides.

To the best of our knowledge, only one plectin specific binding small molecule^45^ and a plectin binding peptide discovered by phage display have been reported so far^46^. It is also interesting to note that the plectin binding peptide had no structural similarities to our PCS2 peptoid^46^. Importantly, peptoids, in general, are very easy to synthesize^47^, protease resistant^48^, non-immunogenic^49^, cell permeable^50,51^ and are rich sources of protein-binding ligands^52^. Furthermore, PCS2 can readily be used as a potential tool to isolate CSCs and/or other plectin positive cancer cell sub-populations that are metastasizing (e.g. Table 1). Subpopulations isolated from variety of cancer types can comparatively be studied and characterized for CSC signatures using this technology.

In summary, we used a peptoid OBTC combinatorial cell screen to unbiasedly target a ALDH^+^ subpopulation in a NSCLC which led to the identification of peptoid PCS2, which in turn led to identification of its binding target, plectin which was expressed on the surface of NSCLC tumor cells but not normal lung epithelial cells. Tumor cells isolated with PCS2 or anti-plectin antibodies displayed increased expression of CSC related genes, and plectin knockdown led to dramatically impaired tumor colony formation and migration/invasion. All of these features nominate the plectin as a biomarker of an important subpopulation of NSCLC cells and as a potential therapeutic target.

## Methods

### Compound synthesis

All the amino acids were purchased from EMD Millipore, MA. All the primary amines (for peptoid synthesis), biotin-maleimide, 4-amonibenzophenone from Sigma-Aldrich, MO. O-Benzotriazole-N,N,N′,N′-tetramethyl-uronium-hexafluoro-phosphate (HBTU) and N–Hydroxybenzotriazole.H_2_O (HOBt) were purchased from AnaSpec, CA. Applied Biosystems Voyager DePro MALDI mass spectrometer was used in positive reflector mode to acquire MALDI-TOF mass spectra. Alpha-Cyano-4-hydroxycinnamic acid (Sigma Aldrich, MO) was used as the matrix. HPLC purification was performed in a Waters 1525 Binary HPLC pump connected to Waters 2487 Dual λ absorbance detector using Protein & Peptide C18 300 A°, 22 × 250 mm, 10 micron column from Grace Davison Discovery Sciences. Compound separation was carried out at room temperature using Acetonitrile (ACN; Honeywell, NJ) and water containing 0.1% Trifluoroacetic acid (TFA; Sigma Aldrich, MO).

### Peptoid library

The synthesis of the peptoid library used to identify PCS1 and PCS2 was previously published^6^ and described briefly on Supplementary Fig. S1.

### Synthesis of biotin-PCS1

Novasyn TGR (EMD Millipore - 200 mg) resin was swelled in DMF for 1 h. First, Fmoc-Cys-OH was coupled using standard coupling agents (HBTU/HOBt/DIPEA) for overnight. After removing Fmoc (20% piperidine), next three amino acids Fmoc-Met-OH, Fmoc-D-Lys(Boc)-OH and Fmoc-Val-OH were introduced using the same protocol with 2 hours reaction. Next, five peptoid residues were coupled using the standard two-step peptoid coupling procedure (acylation and amination) under a microwave (1000 W)-assisted synthesis protocol^53^. Beads were treated with 2 M bromoacetic acid (1 mL) and 3.2 M DIC (1 mL), and microwaved at 10% power (2 × 15 s) with gentle shaking in between for 30 s for the acylation step. After washing with DMF, beads were treated with 2M N-Boc-1,4-diaminobutane (1 mL), and microwave coupling was performed as described above. The procedure was repeated again to attach the remaining four residues, methoxyethylamine, N-Boc-1,4-diaminobutane (twice), p-methoxybenzylamine. Finally, beads were treated with a cleavage cocktail of TFA/H2O/tri-isopropylsilane (95%/2.5%/2.5%) for 2 h. The final compound was purified using HPLC and analyzed by MALDI-TOF (Voyager DE Pro, AB Systems, USA). The purified compound was lyophilized to obtain the dry product. Biotin-maleimide was then coupled to this compound (1 M: 1 M ratio) in buffer solution at pH 7 for overnight. The coupled Biotin-PCS1 compound was then purified using HPLC.

### Synthesis of biotin-PCS2

Biotin-PCS2 was synthesized following same procedure as described above, using Fmoc-Cys-OH, Fmoc-Met-OH, Fmoc-D-Lys(Boc)-OH and Fmoc-Lys(Boc)-OH for peptide region and N-Boc-1,4-diaminobutane, (R)-(+)-α-methylbenzylamine, piperonylamine, N-Boc-1,4-diaminobutane and methoxyethylamine for peptoid region.

### Synthesis of biotinylated PCS2-benzophenone

The biotinylated PCS2 was synthesized as described above, except this time using Fmoc-Glu(biotinyl-PEG)-OH (glutamine pre-loaded with biotin, EMD Millipore, MA) directly as the first residue, which enables us to eliminate final maleimide coupling step needed to bring in biotin moiety. Finally, before cleave the compound off the resin, 4-amonibenzophenone was coupled after bromoacylation in the presence of DIPEA and kept on shaker for overnight. Compound cleavage, confirmations and purifications were completed as described in above.

### Synthesis of biotinylated GU40C-benzophenone

Followed the same protocol described **above** to couple Fmoc-Glu(biotinyl-PEG)-OH as first residue and 4-amonibenzophenone as the last residue and the rest of the GU40C compound was synthesized as previously described^4^.

### On-bead two-color (OBTC) binding assay for combinatorial library screen using ALDH^+/−^ H358 cells

Each time the screens were conducted with about 100,000 peptoid library beads synthesized on TentaGel macro-beads (4 × 100,000 for the completion). To begin, 100,000 beads were swelled in DMF overnight. Next day beads were washed three times in RPMI medium (Sigma-Aldrich, MO) with 5% fetal bovine serum (FBS) and equilibrated in the same medium containing 2% Bovine Serum Albumin (BSA, Sigma-Aldrich, MO) for 1 hour in three polypropylene tubes (~33,000 beads/tube). ALDEFluor separated (see Supplementary information) H358 ALDH^+^ and ALDH^−^ cell groups were washed and suspended in RPMI medium with 5% FBS. H358 ALDH^+^ cells were labeled with Qtracker 655 (red color) and H358 ALDH^−^ cells labeled with Qtracker 565 (green color) (see Supplementary Information). Cells were twice washed with RPMI medium with 5% FBS and re-suspended in RPMI media with 5% FBS and 2% BSA (3 mL for each type). Labeled cells were visualized with special long pass DAPI filter of BX-51 fluorescence microscope (Olympus, PA) with a color camera. Both cell groups were mixed thoroughly and pipetted up and down several times to break the clumps. 2 ml of cell suspension mixture was added to each of the three beads containing polypropylene tubes and incubated at room temperature with gentle shaking for 30 minutes (Final cell density for each cell group was 0.5 × 10^6^ and the total cell density was 1 × 10^6^). The beads were gently washed two times with RPMI medium and visualized under the fluorescent microscope using DAPI filter. Single beads containing only red cell bound were isolated manually, stripped off cells and processed for Edman sequencing (Supplementary Table S1, Fig. S1) as previously published^4^.

### Magnetic bead binding assay

Dynabeads™ MyOne™ Streptavidin T1 (Thermo Fisher) were coated with biotinylated PCS1, 2 and control peptoid PC462^6^. Cells were dissociated using Dissociation Buffer or 1x accutase (Sigma). The beads were equilibrated with 2 million cells and incubated for 10 min at RT with gentle shaking. Bead-bound cells and non-bound cells were separated by magnet and counted by Cellometer Mini (Nexcelom). A fraction of each bead-bound sample was then plated for visualization by microscope and then the remaining cells were harvested for RT-qPCR or used in Clonogenicity assay. For the antibody-based magnetic bead binding assay, biotinylated plectin antibody (Bioss Antibodies, Woburn, MA), was used in place of the biotinylated compounds. For the competitive binding assay (Fig. 3E), 1 µg of c-terminal-targeting plectin antibody (ab83497 or ab32528, Abcam, Cambridge, MA) was added to the incubation between beads and cells.

### Colony formation assay

300 cells from each cell group tested were plated in growth media to 35 mm cell culture plates in duplicate and grown for 2 weeks or until colonies were visible by eye. Each plate was stained with Crystal Violet for 5 minutes, before being washed with MilliQ-filtered dH_2_O. Plates were then photographed and colonies were counted using the MOESM software program^54^, and confirmed through microscopic counting.

### On-bead target pull-down

The assay was performed using Dynabeads coated with PCS2 and control GU40C4 (biotinylated and derivatized with benzophenone). H358 cells (10 million) were incubated with those beads for 10 min at room temperature under agitation. After separating the unbound cells magnetically, the beads were washed, and exposed to UV light for 1 hour to crosslink the benzophenone to bound protein targets on the cell surface. The beads were isolated magnetically, resuspended in NP-40 cell lysis buffer (at 4 °C for 20 minutes) with protease inhibitors, separated magnetically from the lysate, resuspended in 1% SDS and boiled for 10 minutes to denature cross-linked proteins and release from the bead through streptavidin denaturing. The released proteins were run in SDS-PAGE gel electrophoresis alongside the control-compound fraction and the H358 whole cell lysate. Control samples includes non-binding compound (biotin-GU40C4)-coated beads, just beads incubated in H358 lysate, and a biotin-PCS2-benzophenone competed with 1 µg/mL Plectin antibody (ab83497). The protein ladder used was Spectra Multicolor Broad Range Protein Ladder (Thermo Fisher).

### Target identification

After on-bead target pull-down, the gel was run and silver stained, following the established protocol^55^. All steps were followed according to the protocol, except incubation with the developing solution required less than 2 minutes before the bands became clearly visible. Once bands were visible, the bands were extracted and submitted to the Proteomics and Metabolomics Facility at MD Anderson Cancer Center for standard mass spectrometric protein sequencing analysis.

### On-bead target pull-down and western blotting

On-bead target pull-down and gel running was performed as described above and the proteins were transferred to a nitrocellulose membrane. The membrane was then blotted and visualized using the ScanLater Western Blot Kit (Molecular Devices, Sunnyvale, CA) as follows: the membrane is blocked for 1 hour in the blocking buffer, then, separately, 1 µg/ml anti-plectin (ab83497, Abcam), 1:100 anti-MYH9 (sc-98978, Santa Cruz Biotech, Santa Cruz, CA), or 1:100 anti-AHNAK (sc-98373) was added and incubated for 2 hours to overnight. After 5 minutes, the membrane is washed with the wash buffer and is incubated in 1:5000 Eu-labelled anti-rabbit antibody in 1x blocking buffer for 1 hour. The membrane is then washed, and visualized using the SpectraMax i3 spectrophotometer (Molecular Devices). The protein ladder used was Precision Plus Protein Dual Color ladder (Bio-Rad).

### Cell surface fraction western blotting

This was carried out using the Pierce Cell Surface Protein Isolation Kit (Thermo Fisher), following the manufacturer’s protocol and followed up by western blot analysis. The protein ladder used was Spectra Multicolor Broad Range Protein Ladder (Thermo Fisher).

### Patient survival analysis

Survival analysis was based on 1926 lung cancer patients and the online survival software kmplot (www.kmplot.com) (Gyorffy B *et al*., *PLoS One* **8**, e82241, 2013). Plectin probe with Affimetrix ID of 216971_s_st was used in the analysis. The split of high and low expression patients was auto selected in the program using the best cutoff value.

### RNAseq expression and mutation

RNAseq expression and mutation data were generated as previously described (McMillan *et al*., Cell 173, 864–878, 2018).

### Statistical analysis

Where appropriate, p values were calculated by two-tailed t-test using the GraphPad Prism 7 software (GraphPad Software).

## Supplementary information

Supplementary information
