# Supplementary material for: Unbiased peptoid combinatorial cell screen identifies plectin protein as a potential biomarker for lung cancer stem cells
Source: Sci Rep. 2019 Oct 18;9:14954. doi: 10.1038/s41598-019-51004-3 (PMC6802198; doi:10.1038/s41598-019-51004-3)
Supplement: Supplementary file 1 — Supplementary information [file 41598_2019_51004_MOESM1_ESM.pdf]

Unbiased peptoid combinatorial cell screen identifies plectin protein as a potential biomarker for lung cancer stem cells

Aaron C. Raymond,[1] Boning Gao,[3,4,5] Luc Girard,[3,4,5] John D. Minna,[3,4,5,6] D. Gomika Udugamasooriya,[1,2,*]

* Corresponding author

[1]Department of Pharmacological & Pharmaceutical Sciences, University of Houston, 3455 Cullen Blvd., Houston, TX 77204-5037, USA. [2]Department of Cancer Systems Imaging, MD Anderson Cancer Center, 1881 East Road, Houston, TX 77030-4009, USA.

[3]Hamon Center for Therapeutic Oncology Research, [4]Simmons Comprehensive Cancer Center, Departments of [5]Pharmacology, and [6]Internal Medicine, University of Texas Southwestern Medical Center, 5323 Harry Hines Blvd., Dallas, TX 75390.

Department of Pharmacological & Pharmaceutical Sciences
University of Houston
4849 Calhoun Rd, Health Building 2, Room 7033
Houston, TX 77204-5037
Office: 713.743.6357, Fax: 713.743.0698
E-mail: gomika@uh.edu

Supporting Information

Table of Content

Figure S1. Related to Figure 1. Library structure and hit identification.

Figure S2. Related to Figure 1. Characterization of Peptoid PCS2.

Figure S3. Related to Figure 1. Characterization of Peptoid Biotin-PCS1.

Figure S4. Related to Figure 1. Characterization of Peptoid Biotin-PCS2.

Figure S5. Related to Figure 3. Characterization of Peptoid Biotinylated PCS2-benzophenone.

Figure S6. Related to Figure 5. Plectin knockdown leads to a decrease in mobility and scratch healing.

Figure S7. Related to Figure 6. Plectin expression correlates to poor patient survival in lung cancer.

Figure S8. Related to Figure 3. Original western blots of Figure 3B.

Figure S9. Related to Figure 3. Original western blots of Figure 3C.

Figure S10. Related to Figure 4. Original western blots of Figure 4B.

Table S1 Related to Figure 1: Standardized Edman sequencing retention times for relevant monomeric peptoid and amino acid units.

Proteomics Dataset S1: Related to Figure 3. Proteomics analysis of PCS2 pulldown gel band, 500 kDa size.

Proteomics Dataset S2: Related to Figure 3. Proteomics analysis of PCS2 pulldown gel band, 450 kDa (same pulldown attempt as S1).

Proteomics Dataset S3: Related to Figure 3. Proteomics analysis of PCS2 pulldown gel band, 500 kDa (second pulldown attempt).

Proteomics Dataset S4: Related to Figure 3. Proteomics analysis of PCS2 pulldown gel band, 250 kDa (same pulldown attempt as S3).

Proteomics Dataset S5: Related to Figure 3. Proteomics analysis of PCS2 pulldown gel band (third pulldown attempt).

Additional Experimental procedures.

(A) <u>Core Structure of the Library</u>

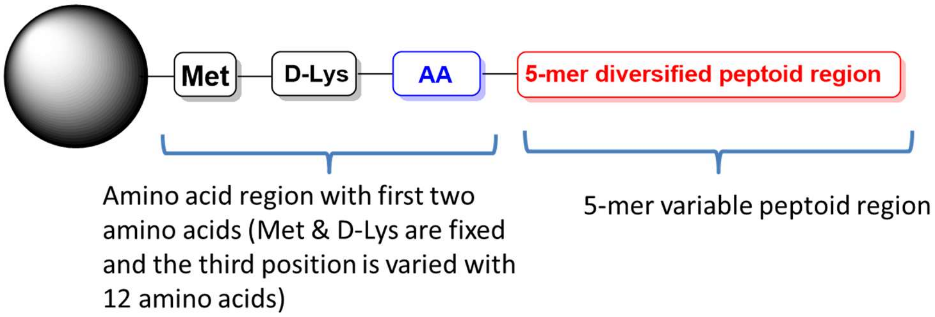

<u>(B) Compound 1: PCS1</u>

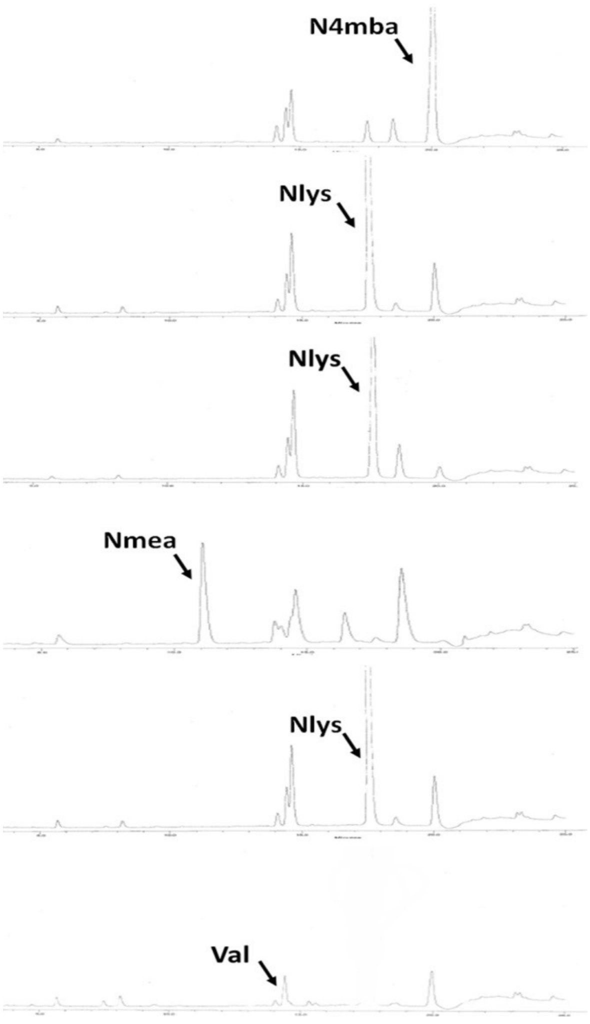

<u>(C) Compound 2: PCS2</u>

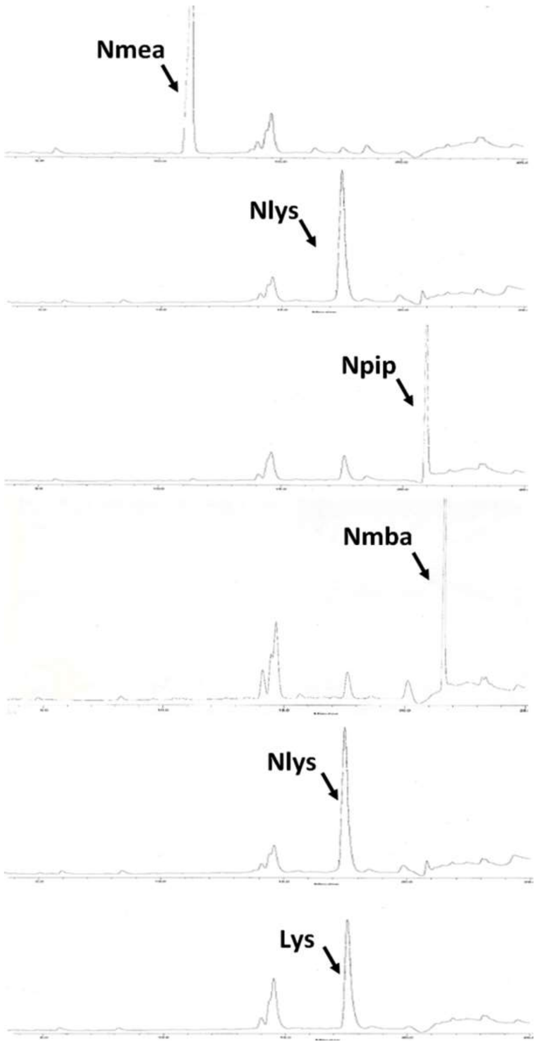

Figure S1: Related to Figure 1. Library structure and hit identification. (A) The core structure of the library: two fixed amino acids, a variable amino acid (3rd position) and five variable peptoid residues (Matharage et al., 2015).**(B & C)** Single bead Edman sequencing spectrum of 'hit' peptoids **(B)** PCS1 and **(C)** PCS2.

S2: Characterization of PCS2

(i)

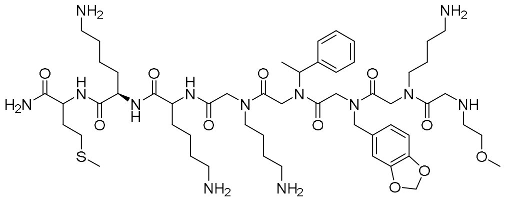

(ii)

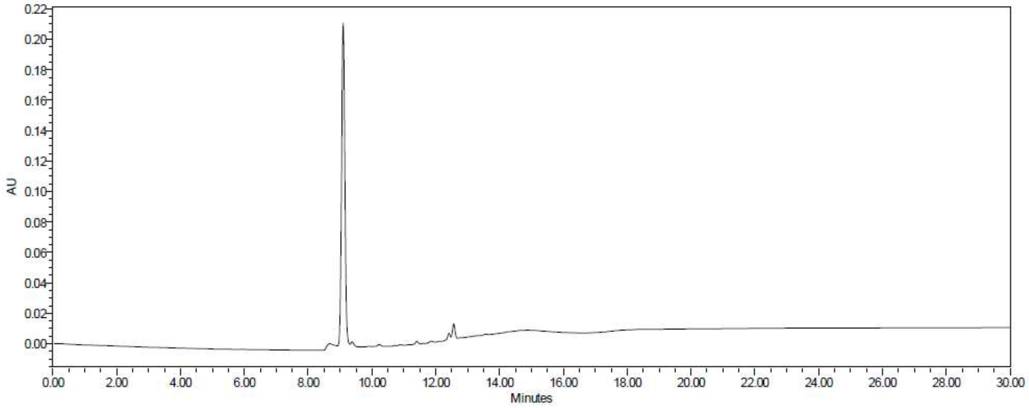

(iii)

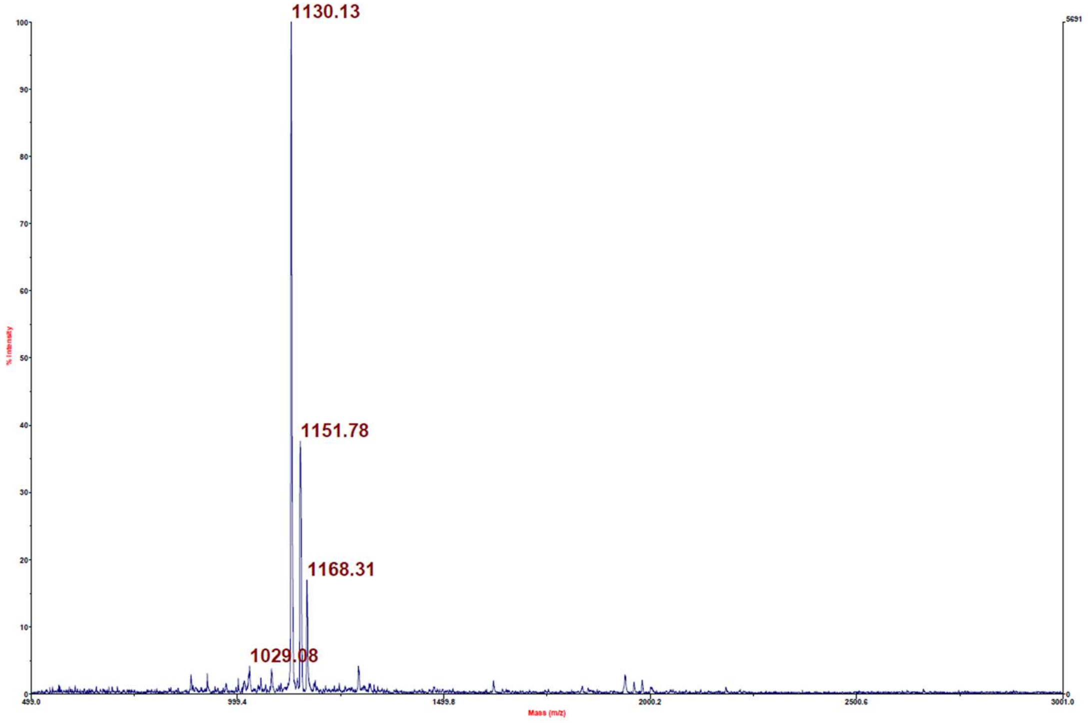

Figure S2 Related to Figure 1: Characterization of Peptoid PCS2 characterized by the following methods: **(i)** Chemical structure, **(ii)** Analytical HPLC **(iii)** MALDI-TOF spectrum.

HRMS ($[M+H]^+$) Calcd. for $C_{54}H_{89}N_{13}O_{11}S$: 1127.65, found: 1130.13

S3: Characterization of Biotin-PCS1

(i)

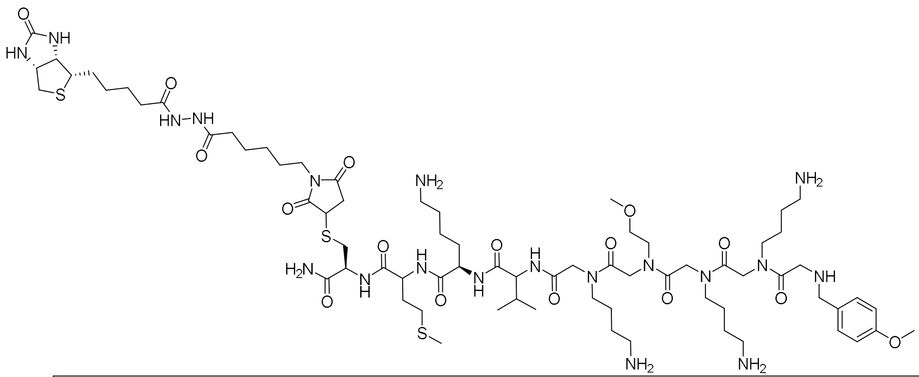

(ii)

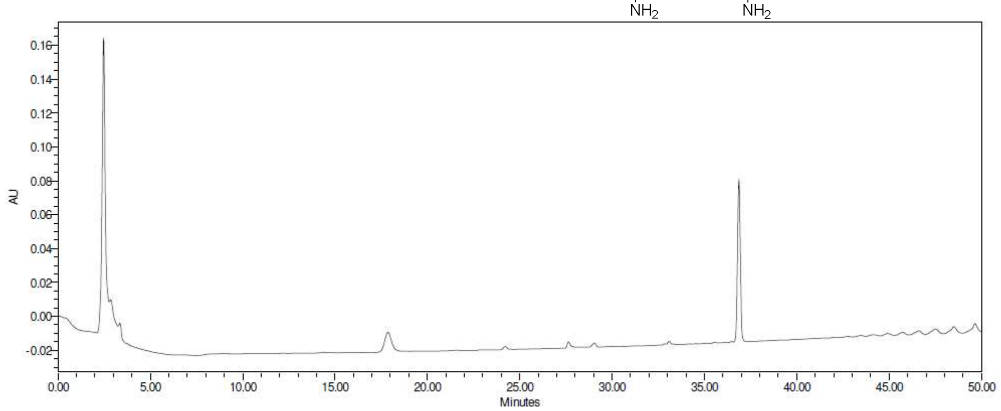

(iii)

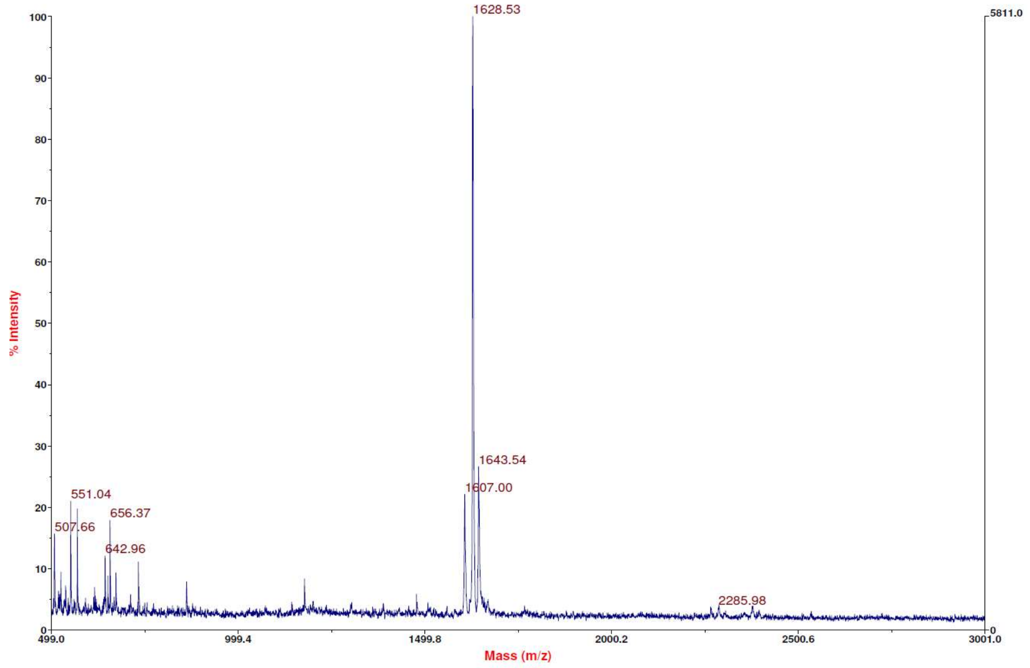

Figure S3 Related to Figure 1: Characterization of Peptoid Biotin-PCS1 characterized by the following methods: **(i)** Chemical structure, **(ii)** Analytical HPLC **(iii)** MALDI-TOF spectrum.

HRMS ([M+H]$^+$) Calcd. for $C_{72}H_{123}N_{19}O_{16}S_3$: 1605.86, found: 1607.00

S4: Characterization of Biotin-PCS2

(i)

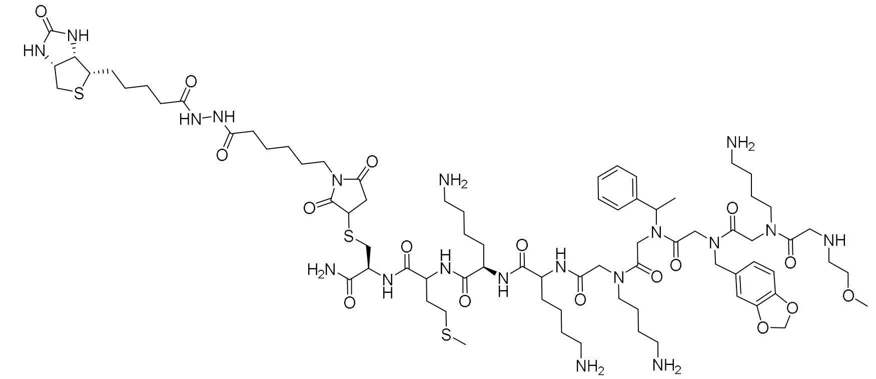

(ii)

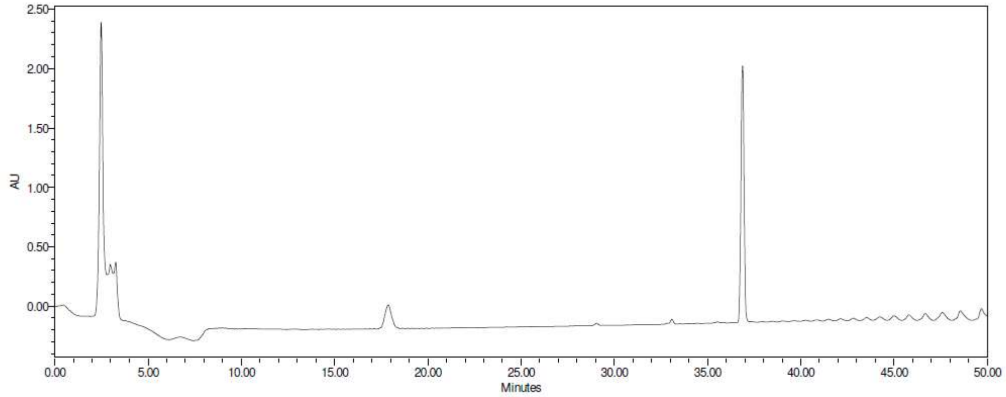

(iii)

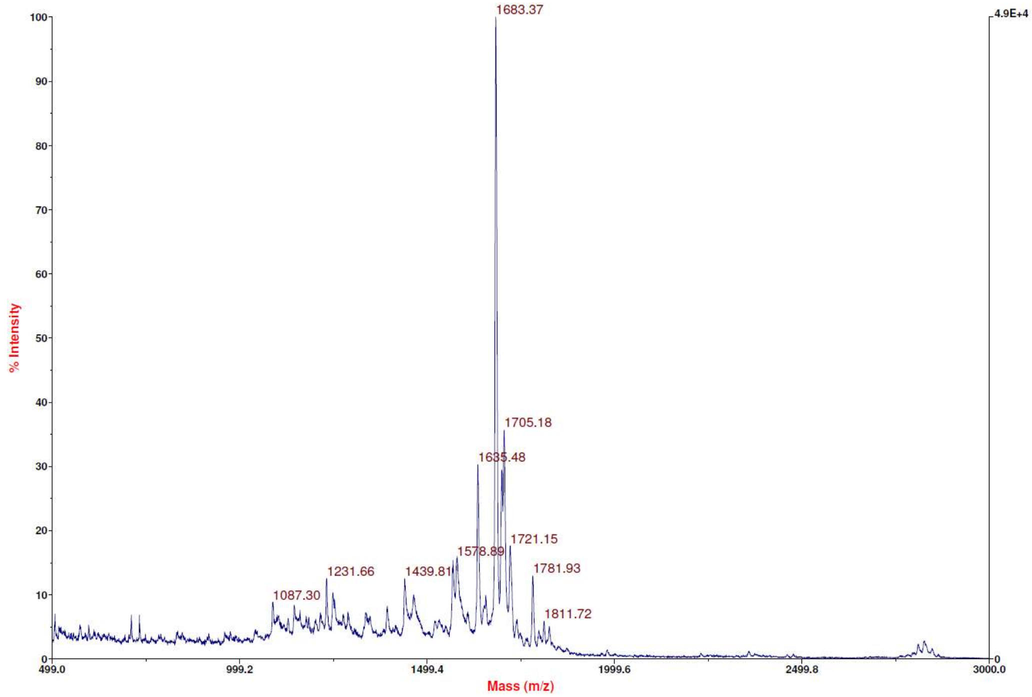

Figure S4 Related to Figure 3: Characterization of Peptoid Biotin-PCS2 characterized by the following methods: **(i)** Chemical structure, **(ii)** Analytical HPLC **(iii)** MALDI-TOF spectrum.

HRMS ($[M+H]^+$) Calcd. for $C_{77}H_{123}N_{19}O_{17}S_3$: 1681.85, found: 1683.37

S5: Characterization of biotinylated PCS2-benzophenone

(i)

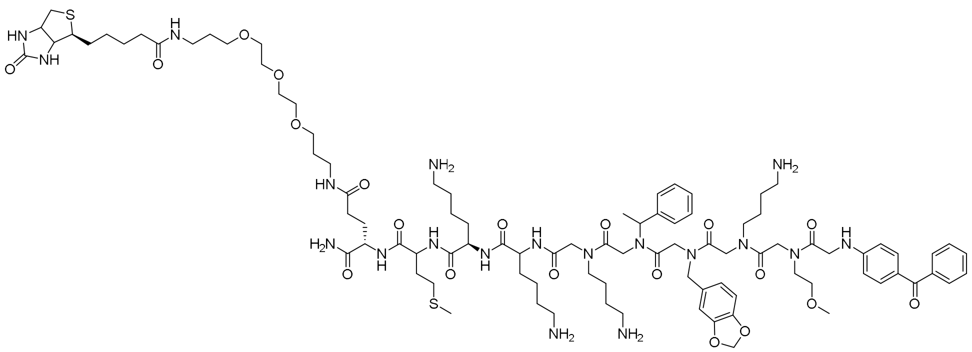

(ii)

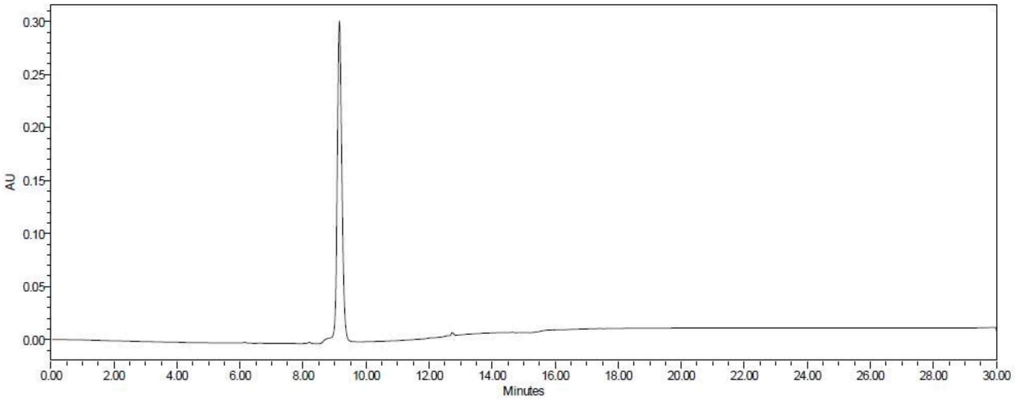

(iii)

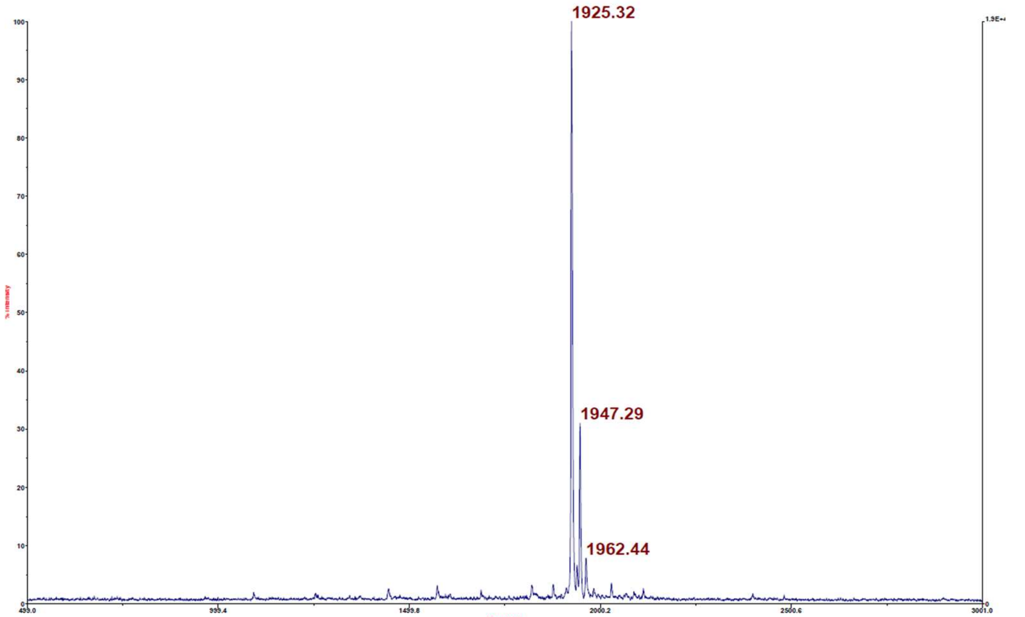

Figure S5 Related to Figure 3: Characterization of Peptoid Biotinylated-PCS2-Benzophenone characterized by the following methods: **(i)** Chemical structure, **(ii)** Analytical HPLC **(iii)** MALDI-TOF spectrum.

HRMS ([M+H]$^+$) Calcd. for $C_{94}H_{143}N_{19}O_{20}S_2$: 1922.02, found: 1925.32

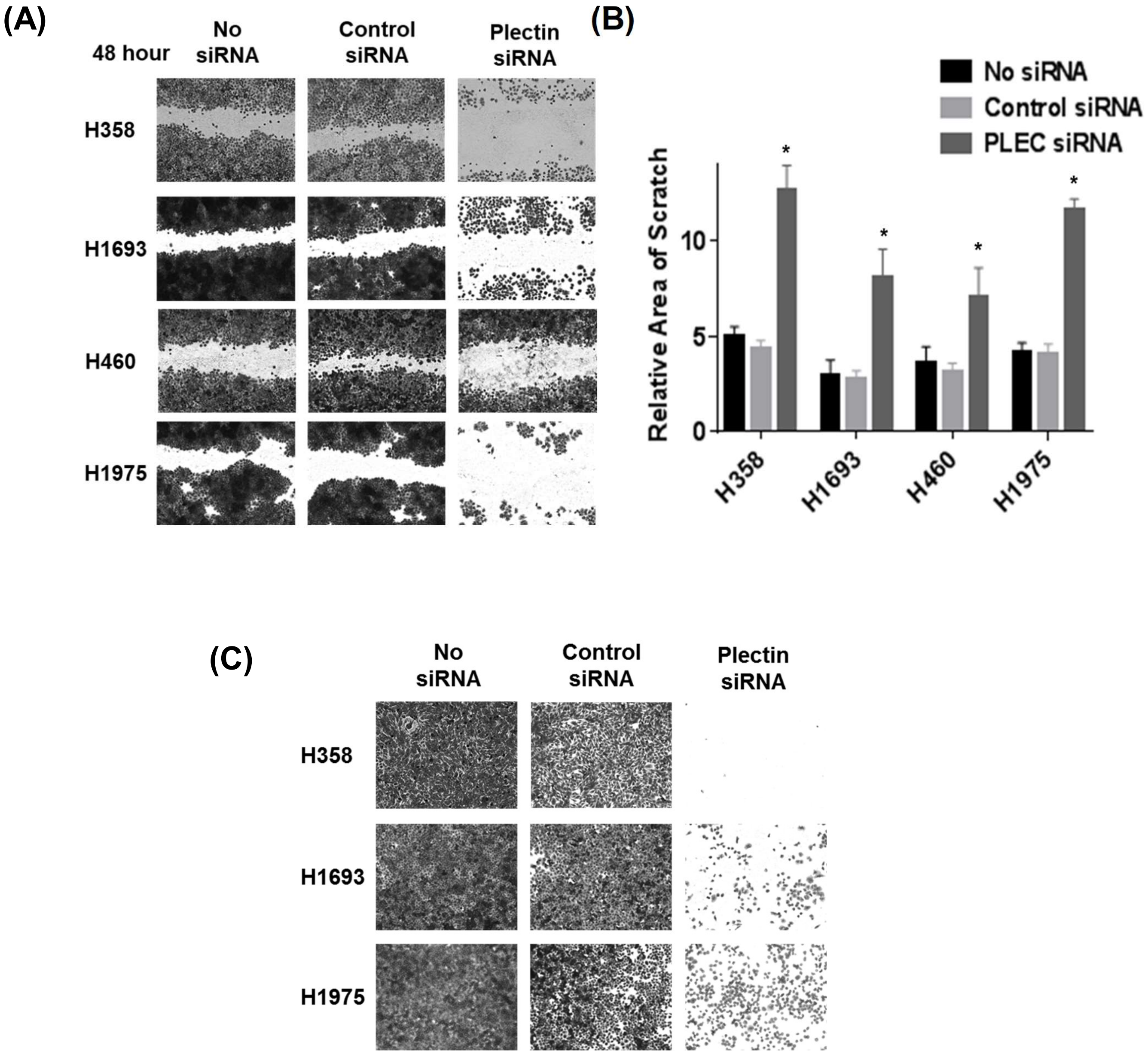

Figure S6 Related to Figure 5: The knockdown of plectin leads to a decrease in mobility and scratch healing in H358, H1693, H460, and H1975 cell lines. **(A & B)** Incubated for 72 hours with siRNA (48 hours before scratch and 24 hours after). **(C)** After 72-hour incubation the plectin siRNA-treated cultures begin to show disruption/instability of the monolayer. Above panels showing 96-hour incubated samples.

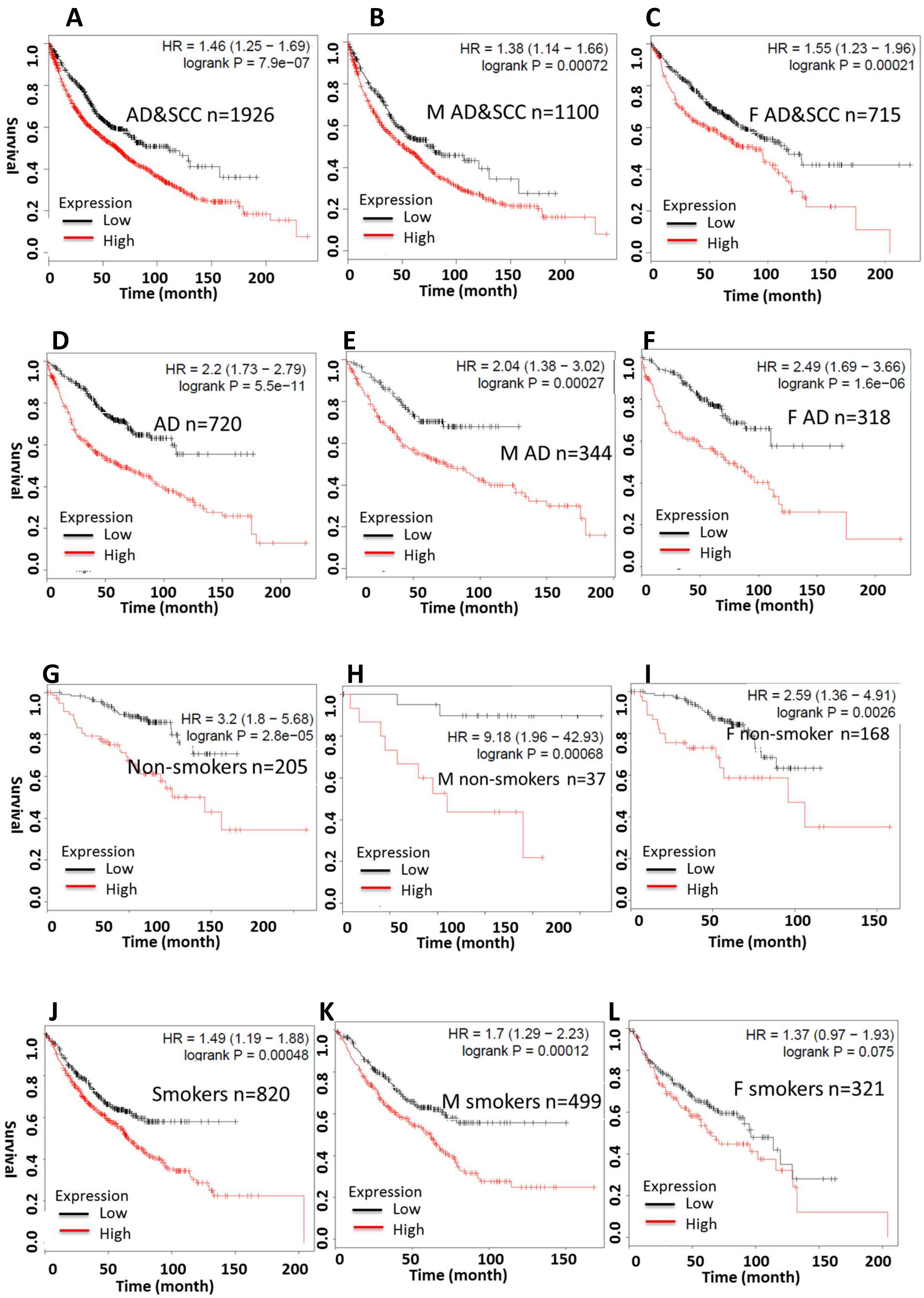
A
HR = 1.46 (1.25 − 1.69)
logrank P = 7.9e−07
AD&SCC n=1926
B
HR = 1.38 (1.14 − 1.66)
logrank P = 0.00072
M AD&SCC n=1100
C
HR = 1.55 (1.23 − 1.96)
logrank P = 0.00021
F AD&SCC n=715
D
HR = 2.2 (1.73 − 2.79)
logrank P = 5.5e−11
AD n=720
E
HR = 2.04 (1.38 − 3.02)
logrank P = 0.00027
M AD n=344
F
HR = 2.49 (1.69 − 3.66)
logrank P = 1.6e−06
F AD n=318
G
HR = 3.2 (1.8 − 5.68)
logrank P = 2.8e−05
Non-smokers n=205
H
HR = 9.18 (1.96 − 42.93)
logrank P = 0.00068
M non-smokers n=37
I
HR = 2.59 (1.36 − 4.91)
logrank P = 0.0026
F non-smoker n=168
J
HR = 1.49 (1.19 − 1.88)
logrank P = 0.00048
Smokers n=820
K
HR = 1.7 (1.29 − 2.23)
logrank P = 0.00012
M smokers n=499
L
HR = 1.37 (0.97 − 1.93)
logrank P = 0.075
F smokers n=321
Survival
Time (month)
Expression
Low
High

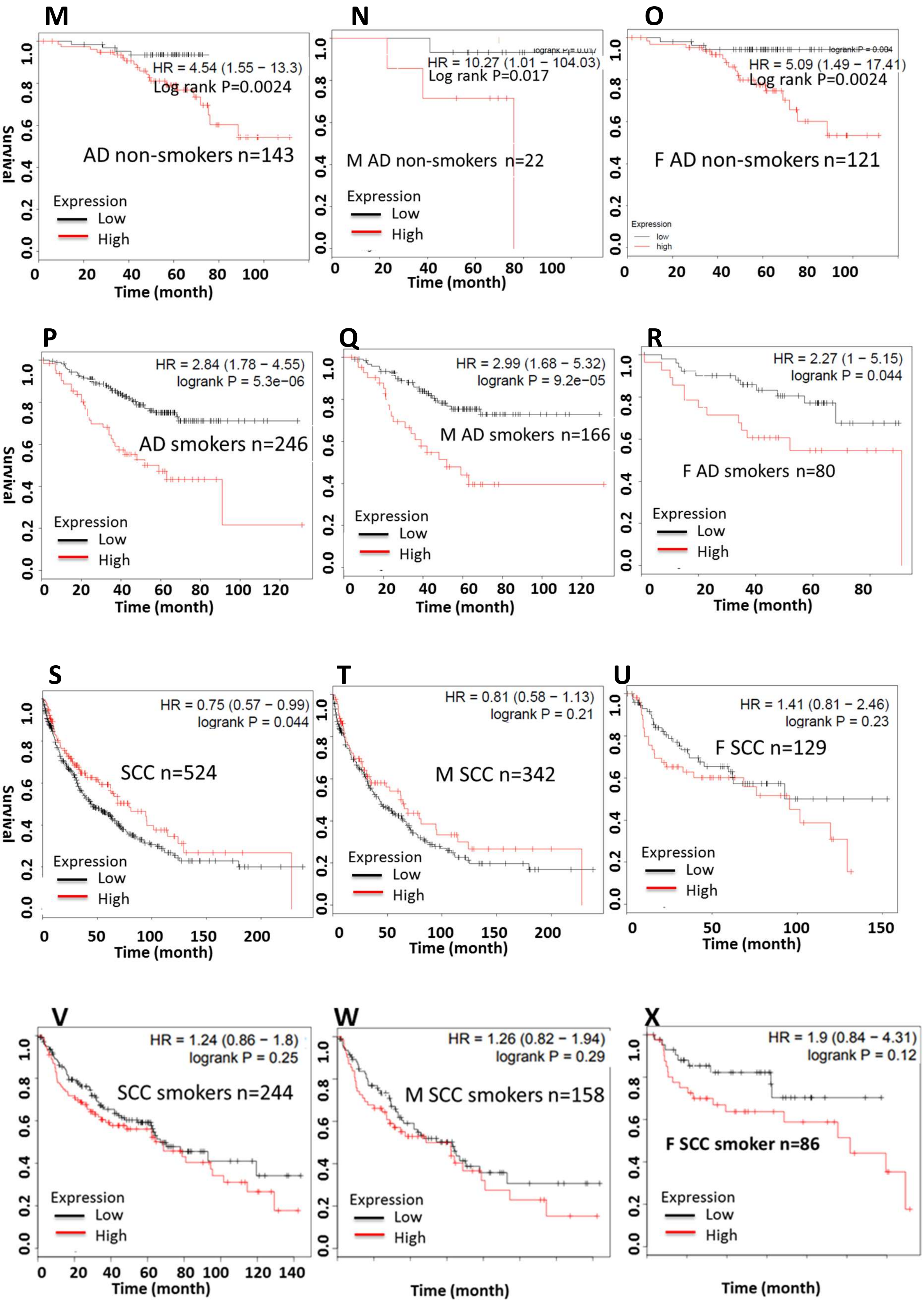
M
HR = 4.54 (1.55 − 13.3)
Log rank P=0.0024
AD non-smokers n=143
N
HR = 10.27 (1.01 − 104.03)
Log rank P=0.017
M AD non-smokers n=22
O
logrank P = 0.004
HR = 5.09 (1.49 − 17.41)
Log rank P=0.0024
F AD non-smokers n=121
P
HR = 2.84 (1.78 − 4.55)
logrank P = 5.3e−06
AD smokers n=246
Q
HR = 2.99 (1.68 − 5.32)
logrank P = 9.2e−05
M AD smokers n=166
R
HR = 2.27 (1 − 5.15)
logrank P = 0.044
F AD smokers n=80
S
HR = 0.75 (0.57 − 0.99)
logrank P = 0.044
SCC n=524
T
HR = 0.81 (0.58 − 1.13)
logrank P = 0.21
M SCC n=342
U
HR = 1.41 (0.81 − 2.46)
logrank P = 0.23
F SCC n=129
V
HR = 1.24 (0.86 − 1.8)
logrank P = 0.25
SCC smokers n=244
W
HR = 1.26 (0.82 − 1.94)
logrank P = 0.29
M SCC smokers n=158
X
HR = 1.9 (0.84 − 4.31)
logrank P = 0.12
F SCC smoker n=86
Survival
Expression
Low
High
Time (month)

Figure S7, Related to Figure 6:

The association of plectin expression and patient overall survival in lung tumor with different subtype, gender and smoking status in KM Plotter analysis. PLEC probe used in the analysis was Affimetrix ID: 216971_s_at. Patient survival on left panels (A, D, G, J, M, P, S and V) is based on male and female combined cases (n=1926). Middle panels (B,E,H,K,N,Q, T and W) and right panels (C,F,I,L,O,R, U and X) are based on analysis in male patients (n=1100) and female patients (n=715) respectively. Panels from top row to bottom row are based on the analysis of different tumor subtype and smoking status as indicated. Red and black bar indicate the high and low expression of plectin respectively. M: male, F: female, AD: adenocarcinoma, SCC: squamous cell carcinoma. HR: hazards ratio. Note: Panel D, P and S are duplication of Fig 6 A, B and C respectively.

Original western blots:

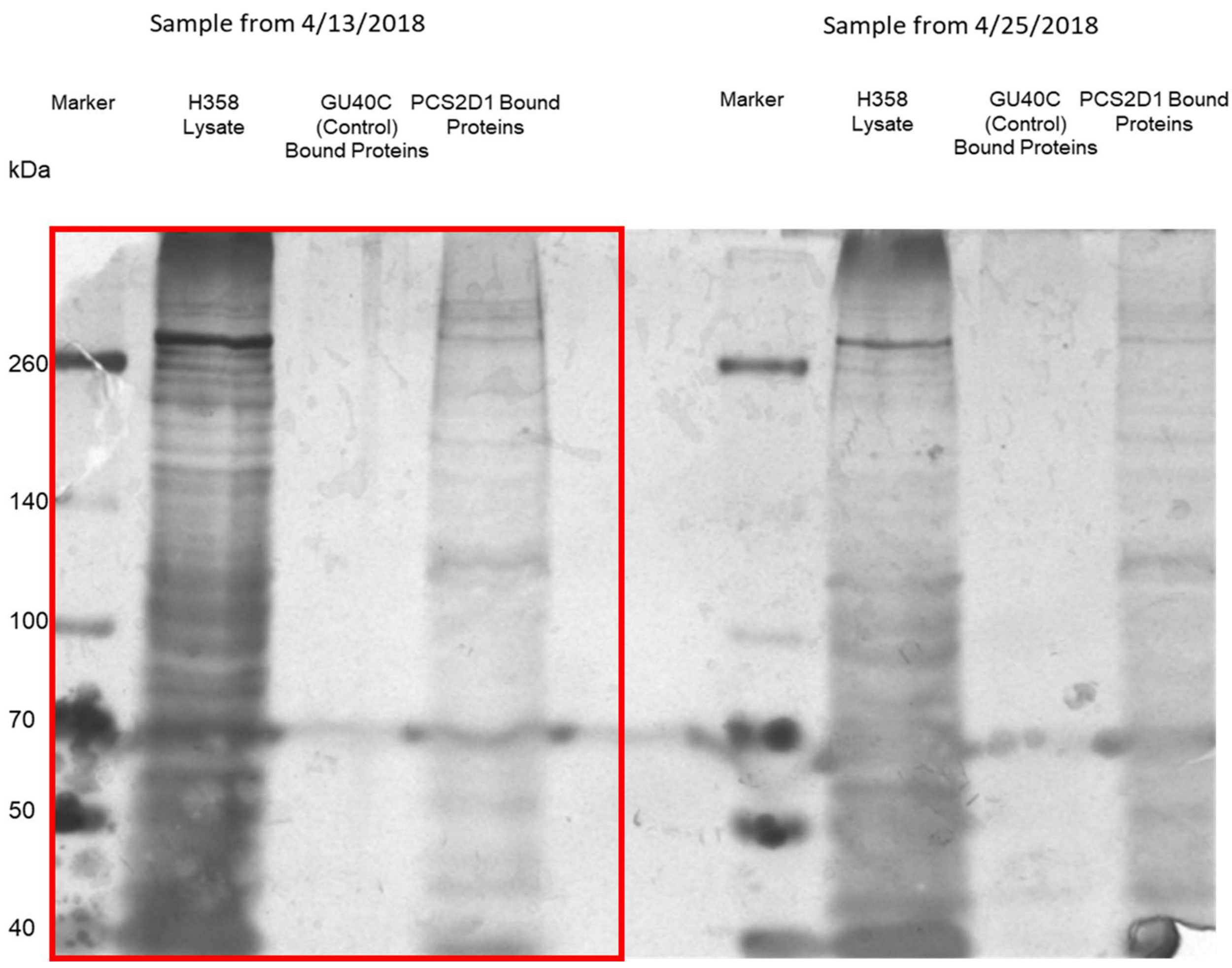

Figure S8. Related to Figure 3. Original western blots of Figure 3B: The PCS2-based magnetic bead pulldown target ID assay (described in **Fig. 3A**) was performed twice within about two weeks time and both samples were run on the same gel. The red highlighted (sample 1) was shown as Figure 3B.

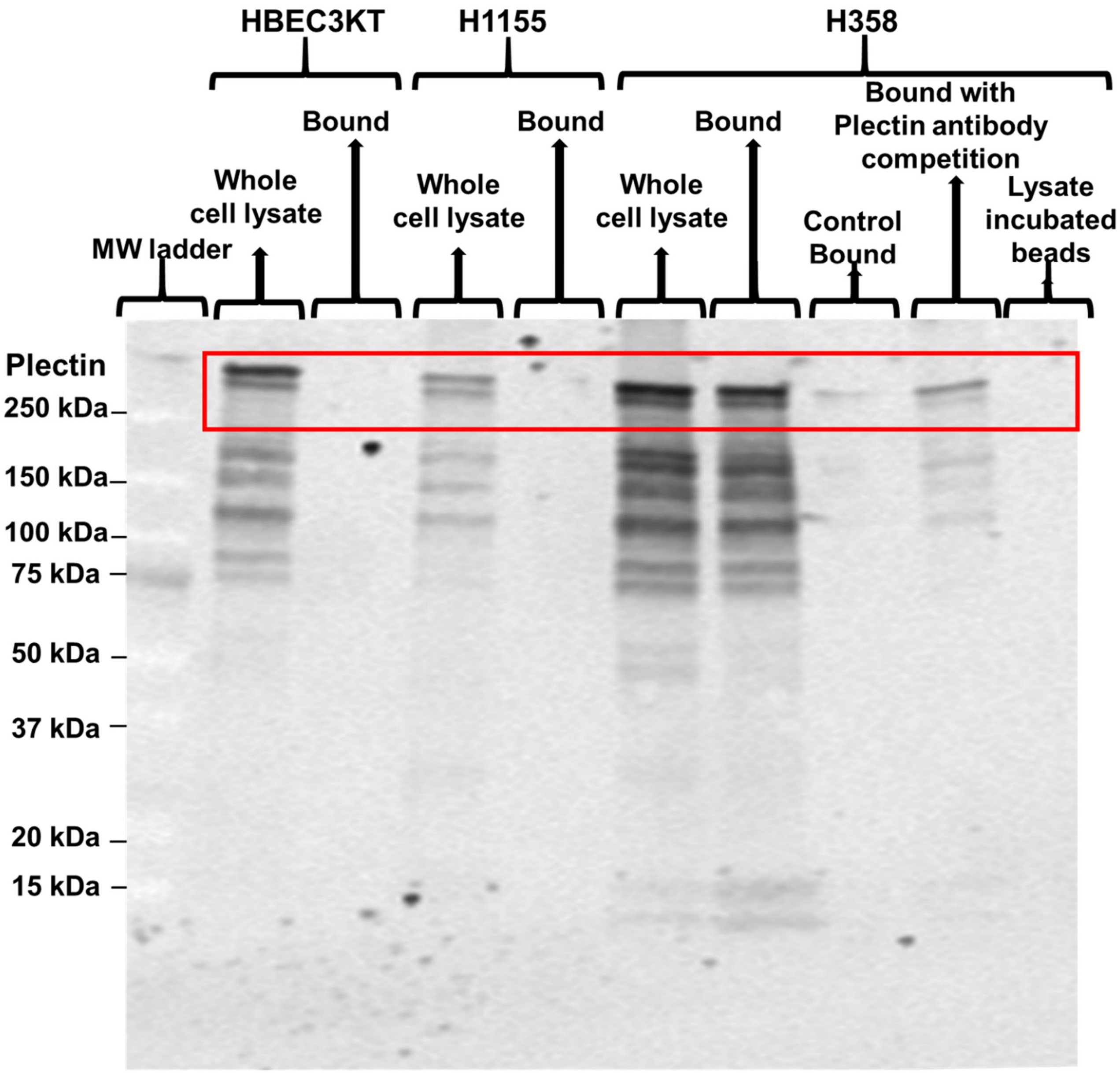

Figure S9. Related to Figure 3. Original western blots of Figure 3C (First panel – Plectin)

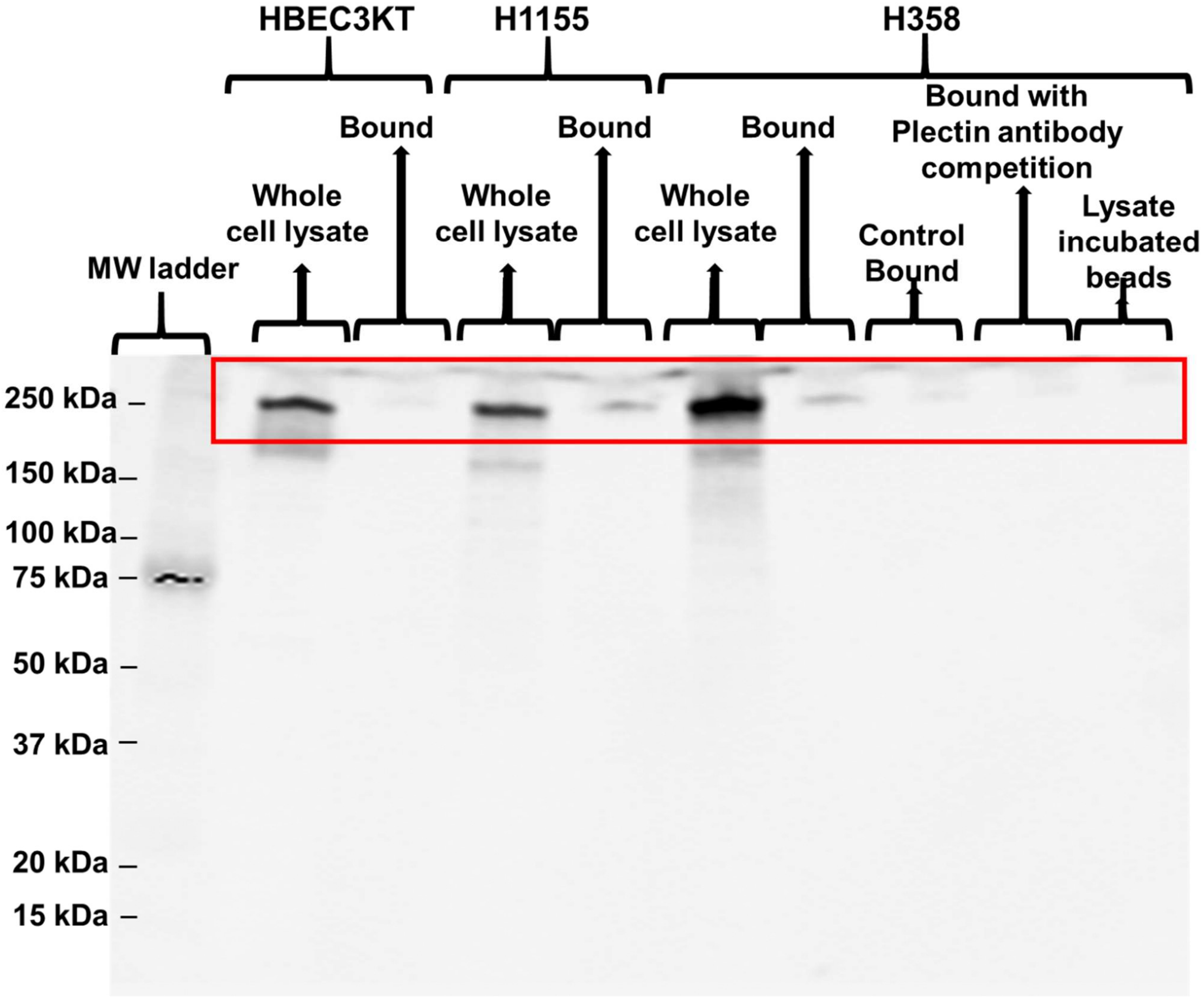

Figure S9. Related to Figure 3. Original western blots of Figure 3C (Second panel – MYH9)

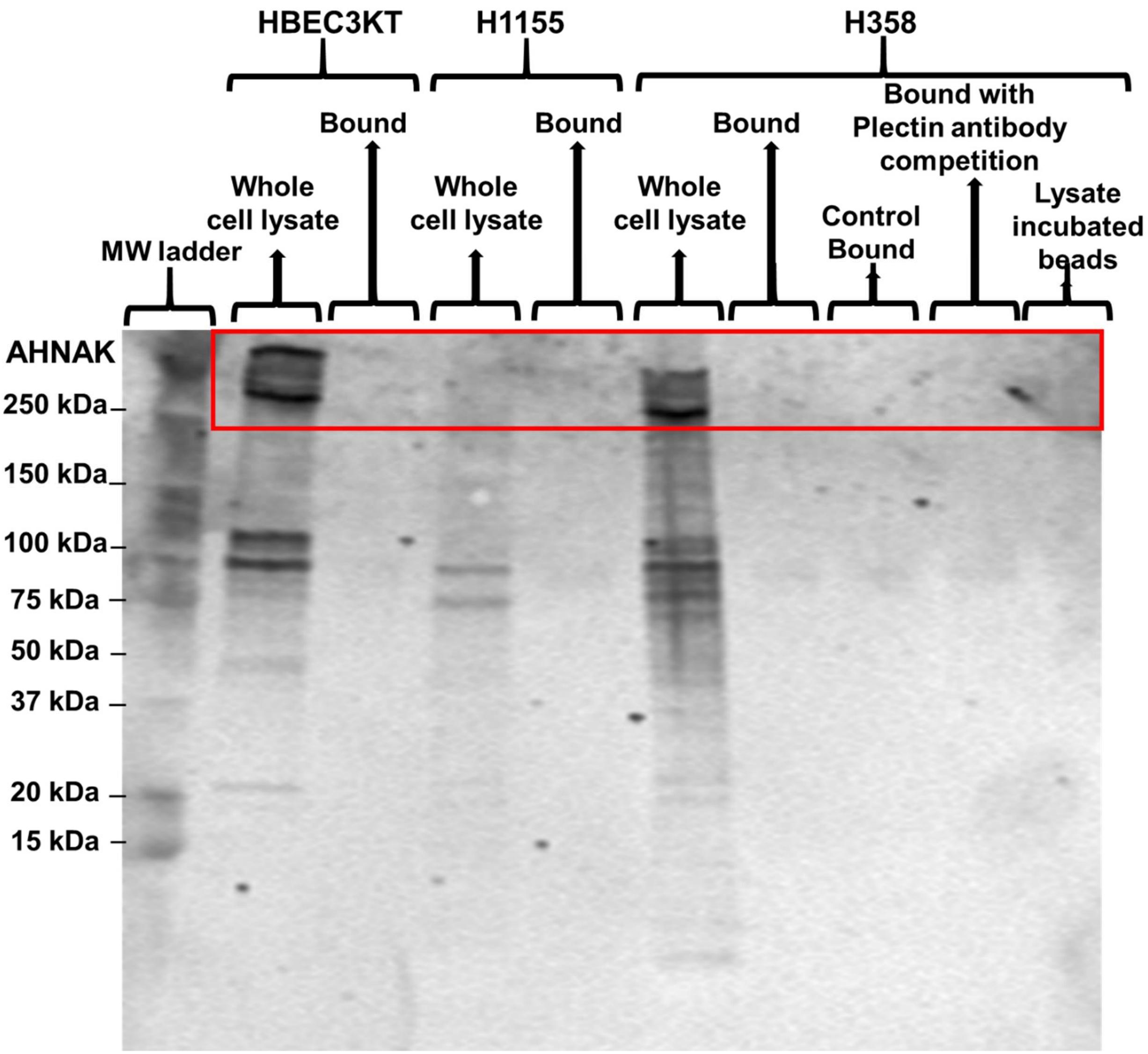

Figure S9. Related to Figure 3. Original western blots of Figure 3C (Third panel – AHNAK)

(A) **Both cell surface and cytosolic fractions in one gel (with plectin antibody):**

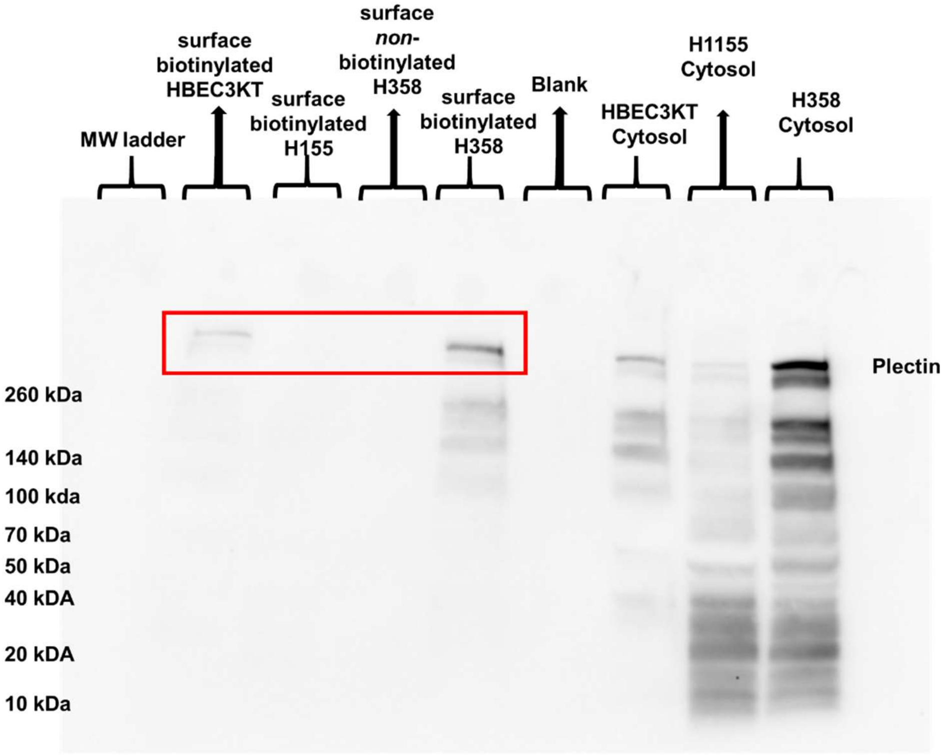

Fig. 4B (Upper panel 1)

(B) **Both cell surface and cytosolic fractions in one gel (with Beta-Actin antibody):**

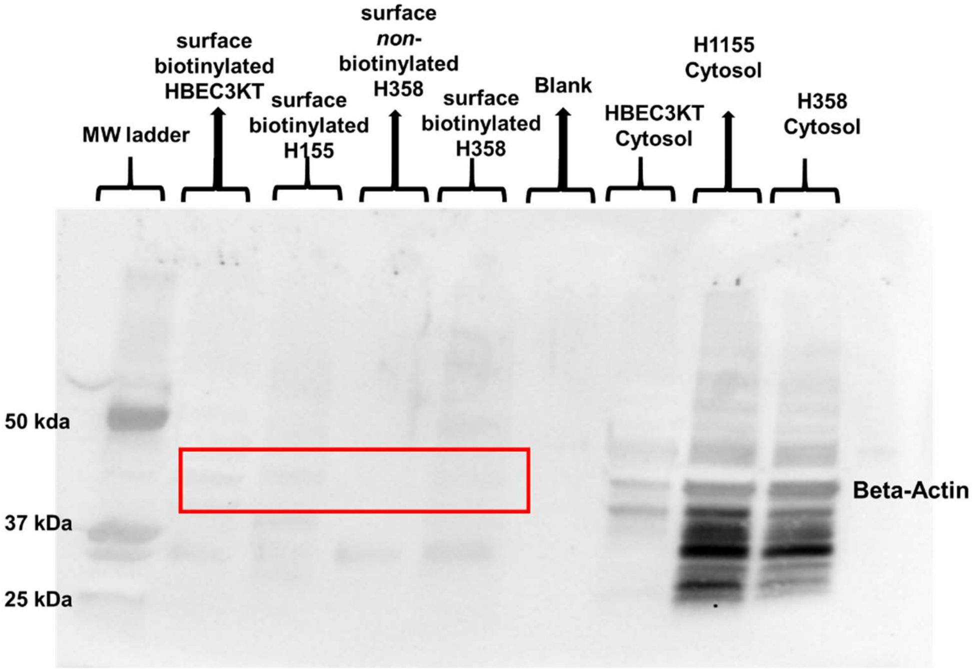

Fig. 4B (Upper panel 2)

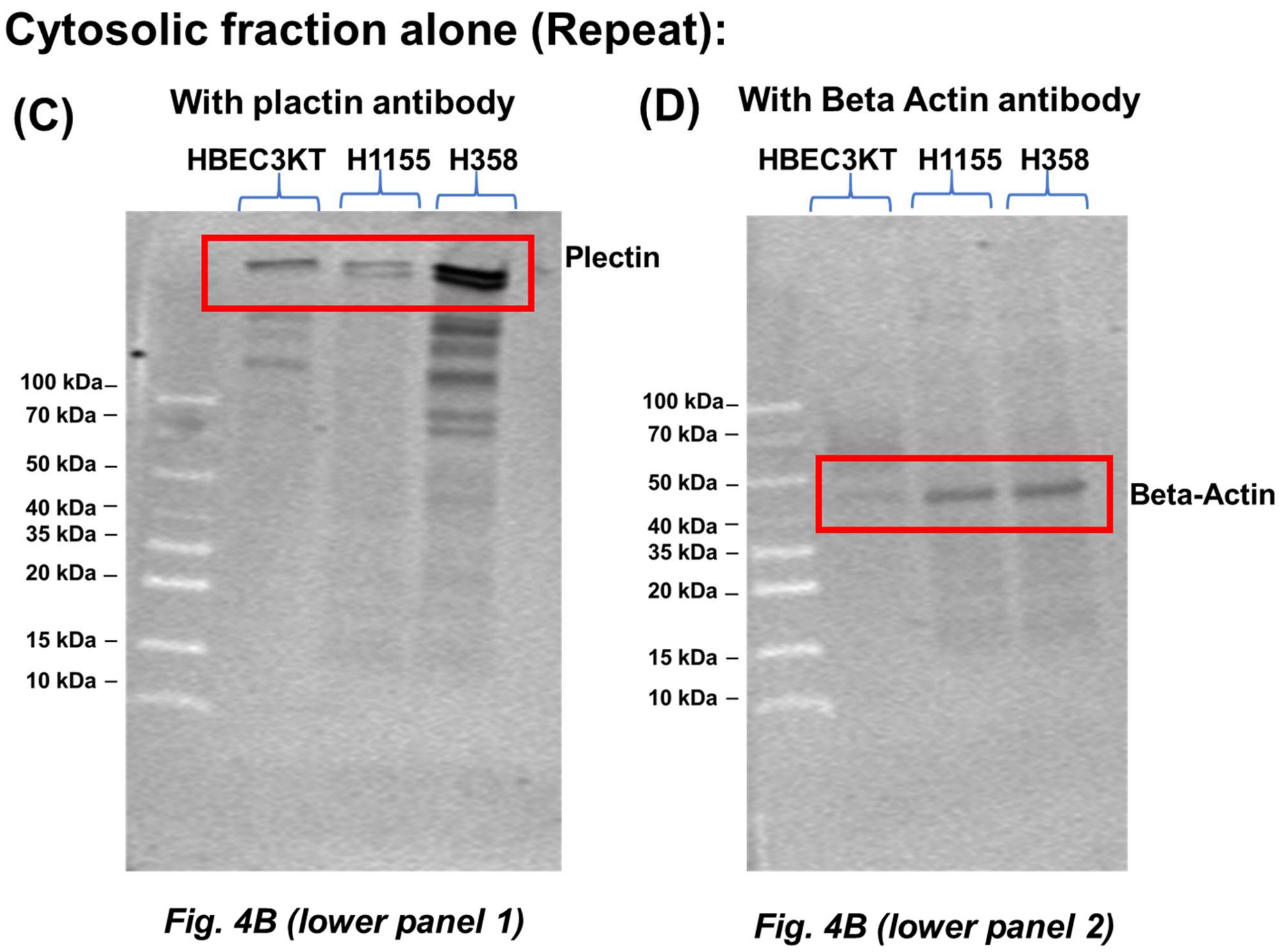

Figure S10. Related to Figure 4. Original western blots of Figure 4B: (A) Both cell surface and cytosolic fractions were run on one gel and developed using plectin antibody. **(B)** Both cell surface and cytosolic fractions were run on one gel and developed using Beta-Actin antibody. **(C)** Cytosolic fraction was repeated with plectin antibody, **(D)** Cytosolic fraction was repeated with Beta actin antibody (Note: each of the western blots were repeated two more rounds and data not shown). Figure 4B was constructed using the cropped areas from **(A)-(D)** blots.

The MW ladders used were Spectra™ Multicolor Broad Range Protein Ladder (Thermo Fisher) and the Precision Plus Protein Dual Color ladder (Bio-Rad).

Table S1 Related to Figure 1: Standardized Edman sequencing retention times for relevant monomeric peptoid and amino acid units.

**Residue**	**structure**	**Standardized Edman sequencing retention time (min)**
Methoxyethylamine (Nmea)	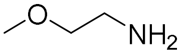	11.3
1,4-Diaminobutane (Nlys)	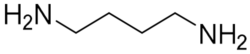	17.6
4-Methoxybenzylamine (N4mba)	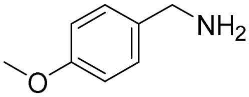	20.1
Piperonylamine (Npip)	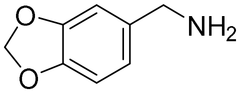	21.0
(R)-Methylbenzylamine (Nmba)	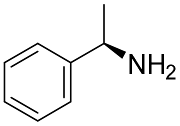	21.8
Amino acid - Lysine	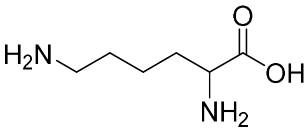	17.2
Amino acid - Valine	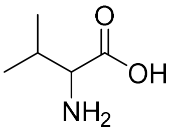	13.9

Proteomics sequencing data 1:

Mascot Search Results

User : Discoverer_Orbitrap Fusion
Search title : 051618ncs-GU818-PCS2D1-1_Node:2
MS data file : File Name: 051618ncs-GU818-PCS2D1-1.raw
Database : SwissProt 2018_02 (556825 sequences; 199652254 residues)
Taxonomy : Homo sapiens (human) (20317 sequences)
Timestamp : 17 May 2018 at 01:13:41 GMT
Enzyme : Trypsin
Variable modifications : Carbamidomethyl (C),Oxidation (M),Gln->pyro-Glu (N-term Q),Trioxidation (C)
Mass values : Monoisotopic
Protein Mass : Unrestricted
Peptide Mass Tolerance : ± 10 ppm
Fragment Mass Tolerance: ± 0.5 Da
Max Missed Cleavages : 2
Instrument type : ESI-TRAP
Number of queries : 1595

Protein hits :
PLEC_HUMAN Plectin OS=Homo sapiens OX=9606 GN=PLEC PE=1 SV=3
FLNB_HUMAN Filamin-B OS=Homo sapiens OX=9606 GN=FLNB PE=1 SV=2
FLNA_HUMAN Filamin-A OS=Homo sapiens OX=9606 GN=FLNA PE=1 SV=4
TPR_HUMAN Nucleoprotein TPR OS=Homo sapiens OX=9606 GN=TPR PE=1 SV=3
KI67_HUMAN Proliferation marker protein Ki-67 OS=Homo sapiens OX=9606 GN=MKI67 PE=1 SV=2
NEST_HUMAN Nestin OS=Homo sapiens OX=9606 GN=NES PE=1 SV=2
UTP20_HUMAN Small subunit processome component 20 homolog OS=Homo sapiens OX=9606 GN=UTP20 PE=1 SV=3
K2C1_HUMAN Keratin, type II cytoskeletal 1 OS=Homo sapiens OX=9606 GN=KRT1 PE=1 SV=6
K1C10_HUMAN Keratin, type I cytoskeletal 10 OS=Homo sapiens OX=9606 GN=KRT10 PE=1 SV=6
FINC_HUMAN Fibronectin OS=Homo sapiens OX=9606 GN=FN1 PE=1 SV=4
ALBU_HUMAN Serum albumin OS=Homo sapiens OX=9606 GN=ALB PE=1 SV=2
K22E_HUMAN Keratin, type II cytoskeletal 2 epidermal OS=Homo sapiens OX=9606 GN=KRT2 PE=1 SV=2
ELYS_HUMAN Protein ELYS OS=Homo sapiens OX=9606 GN=AHCTF1 PE=1 SV=3
K1C9_HUMAN Keratin, type I cytoskeletal 9 OS=Homo sapiens OX=9606 GN=KRT9 PE=1 SV=3
RBP2_HUMAN E3 SUMO-protein ligase RanBP2 OS=Homo sapiens OX=9606 GN=RANBP2 PE=1 SV=2
ZN638_HUMAN Zinc finger protein 638 OS=Homo sapiens OX=9606 GN=ZNF638 PE=1 SV=2
CHD4_HUMAN Chromodomain-helicase-DNA-binding protein 4 OS=Homo sapiens OX=9606 GN=CHD4 PE=1 SV=2
ADT3_HUMAN ADP/ATP translocase 3 OS=Homo sapiens OX=9606 GN=SLC25A6 PE=1 SV=4
MDC1_HUMAN Mediator of DNA damage checkpoint protein 1 OS=Homo sapiens OX=9606 GN=MDC1 PE=1 SV=3
H12_HUMAN Histone H1.2 OS=Homo sapiens OX=9606 GN=HIST1H1C PE=1 SV=2
PCM1_HUMAN Pericentriolar material 1 protein OS=Homo sapiens OX=9606 GN=PCM1 PE=1 SV=4
PWP2_HUMAN Periodic tryptophan protein 2 homolog OS=Homo sapiens OX=9606 GN=PWP2 PE=2 SV=2
ADT2_HUMAN ADP/ATP translocase 2 OS=Homo sapiens OX=9606 GN=SLC25A5 PE=1 SV=7
RPB1_HUMAN DNA-directed RNA polymerase II subunit RPB1 OS=Homo sapiens OX=9606 GN=POLR2A PE=1 SV=2
DIDO1_HUMAN Death-inducer obliterator 1 OS=Homo sapiens OX=9606 GN=DIDO1 PE=1 SV=5
EF1A1_HUMAN Elongation factor 1-alpha 1 OS=Homo sapiens OX=9606 GN=EEF1A1 PE=1 SV=1
RS27A_HUMAN Ubiquitin-40S ribosomal protein S27a OS=Homo sapiens OX=9606 GN=RPS27A PE=1 SV=2

H4_HUMAN Histone H4 OS=Homo sapiens OX=9606 GN=HIST1H4A PE=1 SV=2
H2A1A_HUMAN Histone H2A type 1-A OS=Homo sapiens OX=9606 GN=HIST1H2AA PE=1 SV=3
RIF1_HUMAN Telomere-associated protein RIF1 OS=Homo sapiens OX=9606 GN=RIF1 PE=1 SV=2
RRP5_HUMAN Protein RRP5 homolog OS=Homo sapiens OX=9606 GN=PDCD11 PE=1 SV=3
BAZ2A_HUMAN Bromodomain adjacent to zinc finger domain protein 2A OS=Homo sapiens OX=9606 GN=BAZ2A PE=1 SV=4
INP4B_HUMAN Type II inositol 3,4-bisphosphate 4-phosphatase OS=Homo sapiens OX=9606 GN=INPP4B PE=2 SV=4
YLPM1_HUMAN YLP motif-containing protein 1 OS=Homo sapiens OX=9606 GN=YLPM1 PE=1 SV=3
MYOM1_HUMAN Myomesin-1 OS=Homo sapiens OX=9606 GN=MYOM1 PE=1 SV=2
TCOF_HUMAN Treacle protein OS=Homo sapiens OX=9606 GN=TCOF1 PE=1 SV=3
NAR4_HUMAN Ecto-ADP-ribosyltransferase 4 OS=Homo sapiens OX=9606 GN=ART4 PE=2 SV=2
MPCP_HUMAN Phosphate carrier protein, mitochondrial OS=Homo sapiens OX=9606 GN=SLC25A3 PE=1 SV=2
HUWE1_HUMAN E3 ubiquitin-protein ligase HUWE1 OS=Homo sapiens OX=9606 GN=HUWE1 PE=1 SV=3
PCLO_HUMAN Protein piccolo OS=Homo sapiens OX=9606 GN=PCLO PE=1 SV=4
WDR75_HUMAN WD repeat-containing protein 75 OS=Homo sapiens OX=9606 GN=WDR75 PE=1 SV=1
M3K11_HUMAN Mitogen-activated protein kinase kinase kinase 11 OS=Homo sapiens OX=9606 GN=MAP3K11 PE=1 SV=1
JPH1_HUMAN Junctophilin-1 OS=Homo sapiens OX=9606 GN=JPH1 PE=1 SV=2
ABCAC_HUMAN ATP-binding cassette sub-family A member 12 OS=Homo sapiens OX=9606 GN=ABCA12 PE=1 SV=3
ZN217_HUMAN Zinc finger protein 217 OS=Homo sapiens OX=9606 GN=ZNF217 PE=1 SV=1
TEX2_HUMAN Testis-expressed protein 2 OS=Homo sapiens OX=9606 GN=TEX2 PE=1 SV=2
DPYL5_HUMAN Dihydropyrimidinase-related protein 5 OS=Homo sapiens OX=9606 GN=DPYSL5 PE=1 SV=1
EDEM3_HUMAN ER degradation-enhancing alpha-mannosidase-like protein 3 OS=Homo sapiens OX=9606 GN=EDEM3 PE=1 SV=2
TNS2_HUMAN Tensin-2 OS=Homo sapiens OX=9606 GN=TNS2 PE=1 SV=2
H2B1A_HUMAN Histone H2B type 1-A OS=Homo sapiens OX=9606 GN=HIST1H2BA PE=1 SV=3

SwissProt Decoy False discovery rate
Peptide matches above identity threshold 520 60 11.54 %
Peptide matches above homology or identity threshold 590 64 10.85 %

Select Summary Report
Format As Select Summary (protein hits) Help
Significance threshold p< 0.05 Max. number of hits AUTO Show Percolator scores
Standard scoring MudPIT scoring Display non-significant matches Show sub-sets 0
Show pop-ups Suppress pop-ups Require bold red
Preferred taxonomy All entries
Re-Search All queries Unassigned Below homology threshold Below identity threshold

1. PLEC_HUMAN **Mass:** 531466 **Score:** 3835 **Matches:** 143(143) **Sequences:** 134(134) **emPAI:** 2.12
Plectin OS=Homo sapiens OX=9606 GN=PLEC PE=1 SV=3

Query Observed Mr(expt) Mr(calc) ppm Miss Score Expect Rank Unique Peptide
51 **374.1977 746.3809 746.3810 -0.07 0 22 0.032 2 U R.LSAEAEK.V**
94 **390.2240 778.4334 778.4337 -0.44 0 20 0.034 1 U R.LSFSGLR.A**
124 **401.7064 801.3982 801.3981 0.14 0 32 0.0036 1 U K.AEEQAVR.Q**
156 **414.7506 827.4866 827.4865 0.15 0 22 0.041 1 U R.VAQLLER.W**
184 **423.2092 844.4039 844.4039 0.03 0 56 1.7e-005 1 U R.GAQEVGER.L**
194 **425.7369 849.4593 849.4596 -0.38 0 36 0.002 1 U R.LDLQYAK.L**
207 **432.7322 863.4499 863.4501 -0.20 0 51 0.0001 1 U K.FAEQTLR.Q**
210 **433.2429 864.4712 864.4705 0.84 0 26 0.016 1 U K.FISETLR.R**

230 437.2249 872.4352 872.4352 0.05 0 44 0.00031 1 U K.QVAEEAAR.L
232 437.2556 872.4966 872.4967 -0.13 0 41 0.00085 1 U R.LTVNEAVK.E
238 439.2425 876.4705 876.4705 0.03 0 24 0.044 1 U K.ATVSAPFGK.F
255 445.7236 889.4326 889.4327 -0.10 0 45 0.00038 1 U R.MQEEVVR.R
264 451.2587 900.5028 900.5029 -0.07 0 50 0.00014 1 U K.LAAIGEATR.L
265 451.7507 901.4868 901.4869 -0.05 0 43 0.00069 1 U K.GLVEDTLR.Q
266 451.7507 901.4869 901.4869 0.02 0 47 0.00029 1 U R.LTVDEAVR.A
273 455.7953 909.5761 909.5760 0.19 0 27 0.002 1 U K.AVVQLKPR.H
276 458.2740 914.5335 914.5338 -0.24 0 43 0.00055 1 U K.LLLWSQR.M
278 459.7558 917.4971 917.4971 0.04 0 49 0.00015 1 U R.VPVDVAYR.R
279 460.2402 918.4658 918.4658 0.05 0 37 0.0034 1 U R.TELATQEK.V
304 472.7434 943.4723 943.4723 -0.01 0 47 0.00014 1 U K.ELAQEQAR.R
308 473.2353 944.4560 944.4563 -0.27 0 41 0.00054 1 U R.QLAAEEER.R
309 473.2353 944.4561 944.4563 -0.14 0 44 0.00024 1 U R.LQAEEAER.R
315 473.7455 945.4764 945.4767 -0.29 0 39 0.0018 1 U K.SLAQAEAEK.Q
318 475.7927 949.5708 949.5709 -0.05 0 28 0.002 1 U K.LHVAILER.E
329 479.2592 956.5039 956.5039 -0.02 0 54 2.9e-005 1 U R.AQAEQAALR.Q
334 480.7226 959.4307 959.4308 -0.10 0 45 0.00015 1 U R.QAQEEAER.L
339 482.2445 962.4744 962.4743 0.16 0 36 0.0034 1 U R.VQMEELSK.L
342 484.2535 966.4925 966.4923 0.16 0 34 0.0044 1 U R.SWSLATFR.T
343 484.7472 967.4798 967.4797 0.10 0 46 0.00022 1 U K.VSIYEAMR.R
350 488.2224 974.4301 974.4304 -0.31 0 44 0.00021 1 U R.AAEEAEEAR.V
351 488.2588 974.5030 974.5033 -0.24 0 77 2.9e-007 1 U K.DLSELGSVR.T
357 489.7192 977.4239 977.4236 0.34 1 26 0.015 1 U R.RMEEEER.L
365 492.7592 983.5038 983.5036 0.20 0 46 0.00027 1 U R.VPDVQDGVR.A
370 494.2647 986.5147 986.5145 0.27 1 28 0.015 1 U R.GKAEEQAVR.Q
378 495.2359 988.4572 988.4574 -0.14 0 33 0.0037 1 U R.QVQDESQR.K
379 495.2793 988.5440 988.5441 -0.06 0 58 2.4e-005 1 U R.SELELTLGK.L
381 496.7212 991.4279 991.4280 -0.14 0 52 3.3e-005 1 U K.QAADAEMEK.H
384 497.2481 992.4816 992.4814 0.16 0 40 0.0011 1 U R.FLQEEAEK.M
387 497.2903 992.5661 992.5655 0.61 0 58 7.2e-006 1 U R.LSYTQLLR.R
392 501.2667 1000.5189 1000.5189 -0.02 0 48 0.00017 1 U K.AQAEVEGLGK.G
393 501.7643 1001.5139 1001.5141 -0.20 0 67 2.9e-006 1 U R.QAEVELASR.V
400 503.2552 1004.4959 1004.4960 -0.10 0 39 0.0016 1 U R.LVASMEEAR.R
413 508.2802 1014.5458 1014.5458 0.03 0 93 5.1e-009 1 U R.LSVAAQEAAR.L
418 510.7954 1019.5762 1019.5764 -0.14 0 58 1.1e-005 1 U K.LSVYAALQR.Q
425 512.2897 1022.5648 1022.5648 0.01 0 38 0.0012 1 U R.SLSAIYLEK.L
428 515.2822 1028.5498 1028.5502 -0.39 0 77 2.2e-007 1 U K.AELELELGR.I
429 515.3062 1028.5979 1028.5978 0.04 1 25 0.027 1 U R.RLTVNEAVK.E
430 515.7493 1029.4840 1029.4839 0.08 0 52 4.4e-005 1 U R.AQQQAEAER.A
431 516.2722 1030.5299 1030.5294 0.43 1 25 0.045 1 U R.KQAEEIGEK.L
433 516.2903 1030.5660 1030.5658 0.16 0 41 0.0011 1 U R.LLEAAAQSTK.G
442 517.2584 1032.5023 1032.5022 0.07 0 74 5.1e-007 1 U K.MQAVQEATR.L
454 521.7980 1041.5815 1041.5818 -0.30 0 63 4.3e-006 1 U R.ALQALEELR.L
455 521.7983 1041.5821 1041.5818 0.30 0 76 2.1e-007 1 U R.LAAEQELIR.L
458 522.7878 1043.5610 1043.5611 -0.07 1 26 0.029 1 U R.KAALEEVER.L
462 523.7593 1045.5041 1045.5040 0.17 0 74 3.3e-007 1 U K.SLAAEEEAAR.Q
472 526.7636 1051.5127 1051.5121 0.58 0 59 1.6e-005 1 U R.MVEGYQGLR.C
482 530.2750 1058.5354 1058.5356 -0.25 0 72 6e-007 1 U R.GTQGAEEVLR.A
484 530.7614 1059.5083 1059.5084 -0.15 0 37 0.0021 1 U R.VLADPSDDTK.G
493 533.7856 1065.5566 1065.5567 -0.06 0 28 0.016 1 U R.QLAEAHAQAK.A
505 536.7979 1071.5813 1071.5811 0.11 0 75 1e-006 1 U R.LAEVEAALEK.Q
533 545.7725 1089.5305 1089.5302 0.28 0 59 1.9e-005 1 U R.QSSEAEIQAK.A
534 545.7943 1089.5741 1089.5740 0.10 0 84 6.1e-008 1 U R.AEMEVLLASK.A
548 550.7885 1099.5625 1099.5622 0.28 0 77 2.5e-007 1 U R.GGAEGELQALR.A

550 550.8009 1099.5872 1099.5873 -0.04 0 43 0.00066 1 U R.QLLEEELAR.L 549
552 551.2861 1100.5576 1100.5574 0.18 1 33 0.0038 1 U R.LQAEEAERR.L
553 551.2861 1100.5577 1100.5574 0.26 0 46 0.00023 1 U R.QLAEGTAQQR.L
577 555.8113 1109.6080 1109.6080 -0.04 0 57 1.4e-005 1 U K.AQLEPVASPAK.K
580 557.3096 1112.6047 1112.6050 -0.27 1 30 0.0099 1 U R.RAQAEQAALR.Q
582 372.5604 1114.6594 1114.6597 -0.27 1 (39) 0.00073 1 U R.LKTEAEIALK.E
583 558.3373 1114.6600 1114.6597 0.25 1 46 0.00023 1 U R.LKTEAEIALK.E
584 559.2654 1116.5162 1116.5159 0.25 0 53 4e-005 1 U K.QSAEEQAQAR.A
596 565.2960 1128.5775 1128.5775 0.02 0 49 0.00019 1 U K.NLLDEELQR.L
597 565.3019 1128.5892 1128.5887 0.42 0 43 0.00073 1 U R.NLVDNITGQR.L
598 565.3162 1128.6178 1128.6179 -0.15 0 55 5.1e-005 1 U K.TPVEVPVGGFK.G
599 566.2732 1130.5318 1130.5316 0.24 1 39 0.0011 1 U R.RAAEEAEEAR.V
620 572.3039 1142.5932 1142.5931 0.09 0 62 8.5e-006 1 U R.LQAEEVAQQK.S
627 573.7586 1145.5027 1145.5023 0.36 0 (35) 0.0022 1 U R.QLEMSAEAER.L
639 576.2826 1150.5507 1150.5506 0.13 0 28 0.023 1 U R.QYDIDDAIAK.N
652 580.3070 1158.5993 1158.5993 0.04 1 25 0.048 1 U R.DVAEVDTVRR.A
655 580.7376 1159.4607 1159.4604 0.23 0 52 3e-005 1 U R.GYFDEEMNR.V
656 580.7809 1159.5472 1159.5469 0.25 0 59 1.3e-005 1 U R.SDEGQLSPATR.G
660 582.2717 1162.5289 1162.5288 0.10 0 55 2.7e-005 1 U R.QLEMSAEAER.L
663 582.3065 1162.5984 1162.5982 0.13 1 49 0.00022 1 U R.AQFEQLKDGK.T
669 582.7988 1163.5830 1163.5822 0.65 0 60 1.6e-005 1 U R.LTAEDLFEAR.I
678 584.7598 1167.5050 1167.5044 0.51 0 22 0.044 1 U R.SYVDPSTDER.L
680 585.8219 1169.6292 1169.6292 0.07 0 38 0.0018 1 U K.ELIPTEEALR.L
681 586.3321 1170.6497 1170.6496 0.15 0 63 6.7e-006 1 U R.QVEEEILALK.A
688 588.2682 1174.5219 1174.5214 0.45 0 41 0.00057 1 U R.AETEQGEQQR.Q
706 593.8251 1185.6357 1185.6353 0.34 1 38 0.0021 1 U K.RQELEAELAK.V
709 595.8429 1189.6712 1189.6707 0.49 0 69 1.3e-006 1 U R.LLFNDVQTLK.D
710 397.8754 1190.6045 1190.6043 0.10 1 36 0.004 1 U R.FRELAEEAAR.L
733 602.7725 1203.5305 1203.5302 0.23 0 45 0.00025 1 U R.QQQQMEQER.Q
735 603.3166 1204.6186 1204.6187 -0.05 0 42 0.0009 1 U R.EGLTSIEEVTK.N
742 403.8827 1208.6262 1208.6261 0.01 1 34 0.0053 1 U R.RLEEQAAQHK.A
746 605.8249 1209.6353 1209.6353 0.01 0 66 2.8e-006 1 U R.QLQLAQEAAQK.R
747 608.3093 1214.6040 1214.6044 -0.33 0 48 0.00021 1 U R.GLHQSIEEFR.A
750 608.3200 1214.6255 1214.6255 -0.02 0 46 0.00037 1 U R.RPELEDSTLR.Y
759 613.3300 1224.6454 1224.6463 -0.72 0 (54) 4.4e-005 1 U R.QVQVALETAQR.S
763 614.3383 1226.6620 1226.6619 0.08 0 (48) 0.00017 1 U R.QLQLAQEAAQK.R
769 616.3176 1230.6206 1230.6204 0.17 1 64 6.6e-006 1 U R.SKEQAELEAAR.Q
781 620.8120 1239.6093 1239.6095 -0.13 0 38 0.0019 1 U R.LQLEETDHQK.N
786 621.8438 1241.6731 1241.6728 0.22 0 67 2.6e-006 1 U R.QVQVALETAQR.S
789 622.3358 1242.6571 1242.6568 0.24 0 73 7.1e-007 1 U R.SIQEELQQLR.Q
836 642.3149 1282.6153 1282.6153 0.00 0 (47) 0.00025 1 U R.QLAEEDLAQQR.A
842 642.8613 1283.7081 1283.7085 -0.29 0 44 0.00043 1 U R.SLVPAAELLESR.V
848 643.8461 1285.6776 1285.6779 -0.23 0 62 9.7e-006 1 U R.WQAVLAQTDVR.Q
850 644.3488 1286.6831 1286.6830 0.06 0 69 1.9e-006 1 U K.AQVEQELTTLR.L
862 648.8071 1295.5996 1295.5994 0.18 0 59 1.7e-005 1 U K.GGELVYTDSEAR.D
866 650.3198 1298.6251 1298.6255 -0.32 0 34 0.0049 1 U R.QQGLASYDYVR.R
870 650.8283 1299.6421 1299.6419 0.16 0 74 4.6e-007 1 U R.QLAEEDLAQQR.A
894 660.3457 1318.6768 1318.6769 -0.01 0 58 2.4e-005 1 U K.LEQLFQDEVAK.A
936 675.7816 1349.5487 1349.5484 0.21 0 74 1.4e-007 1 U R.DSQDAGGFGPEDR.L
947 679.3520 1356.6895 1356.6885 0.80 0 46 0.00033 1 U R.QEQALLEEIER.H
949 679.8549 1357.6953 1357.6949 0.25 1 47 0.00028 1 U R.LKQSAEEQAQAR.A
962 458.2322 1371.6748 1371.6742 0.42 1 48 0.00023 1 U R.REEAAVDAQQQK.R
965 458.2404 1371.6994 1371.6994 0.06 1 27 0.026 1 U R.QRELAEQELEK.Q
983 694.3502 1386.6858 1386.6851 0.46 1 35 0.0042 1 U R.ARSDEGQLSPATR.G
996 466.9366 1397.7878 1397.7878 0.03 1 30 0.0083 1 U R.LKAEAELLQQQK.E

1013 705.8704 1409.7262 1409.7263 -0.09 0 60 1.3e-005 1 U R.LAQGHTTVDELAR.R
1014 470.9161 1409.7265 1409.7263 0.15 0 (26) 0.033 1 U R.LAQGHTTVDELAR.R
1032 714.9011 1427.7877 1427.7871 0.38 0 36 0.0025 1 U K.IIITVVEEQEQK.G
1053 722.8608 1443.7071 1443.7066 0.37 1 41 0.0013 1 U R.LRAETEQGEQQR.Q
1055 723.4088 1444.8029 1444.8038 -0.58 0 63 5.5e-006 1 U K.LQNVQIALDYLR.H
1068 731.3723 1460.7301 1460.7293 0.52 0 50 0.00019 1 U R.SQVMDEATALQLR.E
1079 735.3472 1468.6799 1468.6794 0.37 0 74 4.4e-007 1 U R.QQEELLAEENQR.L
1086 493.9352 1478.7839 1478.7841 -0.15 0 27 0.025 1 U R.SLQEEHVAVAQLR.E
1091 743.8601 1485.7057 1485.7059 -0.16 0 (61) 1e-005 1 U R.QQEELLAEENQR.L
1120 764.8735 1527.7324 1527.7317 0.42 0 51 0.0001 1 U R.ESADPLGAWLQDAR.R
1127 766.4463 1530.8780 1530.8770 0.70 0 57 1.2e-005 1 U K.VLALPEPSPAAPTLR.S
1141 778.9175 1555.8204 1555.8205 -0.09 0 104 6.1e-010 1 U R.LQEAGILSAEELQR.L
1152 783.9464 1565.8783 1565.8777 0.37 0 92 4.3e-009 1 U R.APVPASELLASGVLSR.A
1159 785.9073 1569.8000 1569.7999 0.11 0 36 0.0032 1 U R.DDGTGQLLLPLSDAR.K
1189 807.4411 1612.8676 1612.8672 0.28 0 74 5e-007 1 U R.LLDAQLSTGGIVDPSK.S
1215 821.4336 1640.8526 1640.8522 0.26 0 40 0.0012 1 U R.GANVIAGVWLEEAGQK.L
1230 554.6322 1660.8748 1660.8784 -2.20 0 (28) 0.02 1 U K.GIYQSLEGAVQAGQLK.V
1231 831.4457 1660.8769 1660.8784 -0.90 0 29 0.016 1 U K.GIYQSLEGAVQAGQLK.V
1255 854.9176 1707.8206 1707.8203 0.20 0 34 0.0064 1 U R.LLDPEDVDVPQPDEK.S
1303 886.9294 1771.8443 1771.8451 -0.42 0 31 0.013 1 U R.DPYSGSTISLFQAMQK.G
1312 892.4633 1782.9120 1782.9112 0.45 0 54 6e-005 1 U R.AALAHSEEVTASQVAATK.T
1335 904.9549 1807.8952 1807.8952 0.01 0 73 8.9e-007 1 U K.VQSGSESVIQEYVDLR.T
1391 628.6654 1882.9744 1882.9748 -0.22 1 44 0.00063 1 U R.EQLRQEQALLEEIER.H
1431 998.0531 1994.0916 1994.0909 0.38 0 27 0.013 1 U K.AGVAAPATQVAQVTLQSVQR.R

Proteomics Dataset S1: Related to Figure 3: Proteomics analysis of PCS2 pulldown gel band at approximately 500 kDa. Mascot analysis lists the top scoring peptides from the search. Majority of the results contain common contaminants proteins such as keratin. Plectin was the potential prediction, both showing a high score and sufficient number of queries sufficient to justify investigating further**.**

Proteomics sequencing data 2:

Mascot Search Results

User : Discoverer_Orbitrap Fusion
Search title : 051818ncs-GU818-PCS2D1-10ulIP-2_Node:2
MS data file : File Name: 051818ncs-GU818-PCS2D1-10ulIP-2.raw
Database : SwissProt 2018_02 (556825 sequences; 199652254 residues)
Taxonomy : Homo sapiens (human) (20317 sequences)
Timestamp : 19 May 2018 at 17:56:11 GMT
Enzyme : Trypsin
Variable modifications : Carbamidomethyl (C),Oxidation (M),Gln->pyro-Glu (N-term Q),Trioxidation (C)
Mass values : Monoisotopic
Protein Mass : Unrestricted
Peptide Mass Tolerance : ± 10 ppm
Fragment Mass Tolerance: ± 0.5 Da
Max Missed Cleavages : 2
Instrument type : ESI-TRAP
Number of queries : 1418

Protein hits :
PLEC_HUMAN Plectin OS=Homo sapiens OX=9606 GN=PLEC PE=1 SV=3
K2C1_HUMAN Keratin, type II cytoskeletal 1 OS=Homo sapiens OX=9606 GN=KRT1 PE=1 SV=6
K1C10_HUMAN Keratin, type I cytoskeletal 10 OS=Homo sapiens OX=9606 GN=KRT10 PE=1 SV=6
K1C9_HUMAN Keratin, type I cytoskeletal 9 OS=Homo sapiens OX=9606 GN=KRT9 PE=1 SV=3
K22E_HUMAN Keratin, type II cytoskeletal 2 epidermal OS=Homo sapiens OX=9606 GN=KRT2 PE=1 SV=2
CO1A1_HUMAN Collagen alpha-1(I) chain OS=Homo sapiens OX=9606 GN=COL1A1 PE=1 SV=5
ALBU_HUMAN Serum albumin OS=Homo sapiens OX=9606 GN=ALB PE=1 SV=2
K1C14_HUMAN Keratin, type I cytoskeletal 14 OS=Homo sapiens OX=9606 GN=KRT14 PE=1 SV=4
CO1A2_HUMAN Collagen alpha-2(I) chain OS=Homo sapiens OX=9606 GN=COL1A2 PE=1 SV=7
K2C5_HUMAN Keratin, type II cytoskeletal 5 OS=Homo sapiens OX=9606 GN=KRT5 PE=1 SV=3
KI67_HUMAN Proliferation marker protein Ki-67 OS=Homo sapiens OX=9606 GN=MKI67 PE=1 SV=2
RGPD1_HUMAN RANBP2-like and GRIP domain-containing protein 1 OS=Homo sapiens OX=9606 GN=RGPD1 PE=2 SV=1
H4_HUMAN Histone H4 OS=Homo sapiens OX=9606 GN=HIST1H4A PE=1 SV=2
LYSC_HUMAN Lysozyme C OS=Homo sapiens OX=9606 GN=LYZ PE=1 SV=1
BRWD3_HUMAN Bromodomain and WD repeat-containing protein 3 OS=Homo sapiens OX=9606 GN=BRWD3 PE=1 SV=2
H2A1A_HUMAN Histone H2A type 1-A OS=Homo sapiens OX=9606 GN=HIST1H2AA PE=1 SV=3
RSSA_HUMAN 40S ribosomal protein SA OS=Homo sapiens OX=9606 GN=RPSA PE=1 SV=4
ZCCHV_HUMAN Zinc finger CCCH-type antiviral protein 1 OS=Homo sapiens OX=9606 GN=ZC3HAV1 PE=1 SV=3
NCOA6_HUMAN Nuclear receptor coactivator 6 OS=Homo sapiens OX=9606 GN=NCOA6 PE=1 SV=3
MEF2A_HUMAN Myocyte-specific enhancer factor 2A OS=Homo sapiens OX=9606 GN=MEF2A PE=1 SV=1
CRY1_HUMAN Cryptochrome-1 OS=Homo sapiens OX=9606 GN=CRY1 PE=1 SV=1
SPTN2_HUMAN Spectrin beta chain, non-erythrocytic 2 OS=Homo sapiens OX=9606 GN=SPTBN2 PE=1 SV=3
RP3A_HUMAN Rabphilin-3A OS=Homo sapiens OX=9606 GN=RPH3A PE=1 SV=1
COSA1_HUMAN Collagen alpha-1(XXVIII) chain OS=Homo sapiens OX=9606 GN=COL28A1 PE=2 SV=2
EDEM3_HUMAN ER degradation-enhancing alpha-mannosidase-like protein 3 OS=Homo sapiens OX=9606 GN=EDEM3 PE=1 SV=2
NMDE2_HUMAN Glutamate receptor ionotropic, NMDA 2B OS=Homo sapiens OX=9606 GN=GRIN2B PE=1 SV=3

SwissProt Decoy False discovery rate
Peptide matches above identity threshold 164 32 19.51 %
Peptide matches above homology or identity threshold 185 35 18.92 %

Select Summary Report
Format As Select Summary (protein hits)
Significance threshold p< 0.05 Max. number of hits AUTO Show Percolator scores
Standard scoring MudPIT scoring Display non-significant matches Show sub-sets 0
Show pop-ups Suppress pop-ups Require bold red
Preferred taxonomy All entries
Re-Search All queries Unassigned Below homology threshold Below identity threshold

1. PLEC_HUMAN Mass: 531466 Score: 1333 Matches: 51(51) Sequences: 50(50) emPAI: 0.51
Plectin OS=Homo sapiens OX=9606 GN=PLEC PE=1 SV=3

Query	Observed	Mr(expt)	Mr(calc)	ppm	Miss	Score	Expect	Rank	Unique	Peptide
168	423.2093	844.4040	844.4039	0.17	0	30	0.0068	1	U	R.GAQEVGER.L
208	437.2249	872.4353	872.4352	0.12	0	26	0.019	1	U	K.QVAEEAAR.L
252	451.2586	900.5026	900.5029	-0.27	0	40	0.0014	1	U	K.LAAIGEATR.L
253	451.7508	901.4870	901.4869	0.15	0	25	0.048	1	U	K.GLVEDTLR.Q
265	459.7559	917.4973	917.4971	0.30	0	36	0.0035	1	U	R.VPVDVAYR.R
322	484.7470	967.4794	967.4797	-0.28	0	49	0.00012	1	U	K.VSIYEAMR.R
329	488.2226	974.4306	974.4304	0.12	0	61	3.7e-006	1	U	R.AAEEAEEAR.V
330	488.2590	974.5034	974.5033	0.19	0	55	4e-005	1	U	K.DLSELGSVR.T
354	495.2793	988.5441	988.5441	0.06	0	49	0.00016	1	U	R.SELELTLGK.L
374	508.2800	1014.5455	1014.5458	-0.26	0	65	3.4e-006	1	U	R.LSVAAQEAAR.L
379	510.7952	1019.5758	1019.5764	-0.50	0	48	0.00011	1	U	K.LSVYAALQR.Q
389	516.2905	1030.5664	1030.5658	0.53	0	54	4.8e-005	1	U	R.LLEAAAQSTK.G
406	523.7593	1045.5041	1045.5040	0.17	0	54	3.8e-005	1	U	K.SLAAEEEAAR.Q
421	530.2748	1058.5351	1058.5356	-0.47	0	69	1.3e-006	1	U	R.GTQGAEEVLR.A
423	530.7617	1059.5088	1059.5084	0.33	0	46	0.0003	1	U	R.VLADPSDDTK.G
441	536.7981	1071.5816	1071.5811	0.47	0	71	2.6e-006	1	U	R.LAEVEAALEK.Q
463	545.7724	1089.5302	1089.5302	0.06	0	26	0.036	1	U	R.QSSEAEIQAK.A
464	545.7947	1089.5748	1089.5740	0.78	0	52	0.00013	1	U	R.AEMEVLLASK.A
481	550.7881	1099.5617	1099.5622	-0.38	0	76	2.9e-007	1	U	R.GGAEGELQALR.A
482	550.8010	1099.5874	1099.5873	0.07	0	40	0.0013	1	U	R.QLLEEELAR.L
483	551.2865	1100.5584	1100.5574	0.93	0	60	1e-005	1	U	R.QLAEGTAQQR.L
500	555.8115	1109.6084	1109.6080	0.29	0	55	3e-005	1	U	K.AQLEPVASPAK.K
519	565.2961	1128.5776	1128.5775	0.12	0	28	0.022	1	U	K.NLLDEELQR.L
520	565.3162	1128.6178	1128.6179	-0.15	0	44	0.0006	1	U	K.TPVEVPVGGFK.G
539	572.3038	1142.5931	1142.5931	-0.01	0	57	2.8e-005	1	U	R.LQAEEVAQQK.S
560	580.7374	1159.4603	1159.4604	-0.08	0	57	7.8e-006	1	U	R.GYFDEEMNR.V
569	582.7986	1163.5827	1163.5822	0.43	0	30	0.014	1	U	R.LTAEDLFEAR.I
571	585.8218	1169.6290	1169.6292	-0.14	0	32	0.0079	1	U	K.ELIPTEEALR.L
588	595.8428	1189.6710	1189.6707	0.27	0	51	7.3e-005	1	U	R.LLFNDVQTLK.D
621	602.7724	1203.5302	1203.5302	0.03	0	23	0.041	1	U	R.QQQMEQER.Q
622	603.3171	1204.6196	1204.6187	0.77	0	53	9.1e-005	1	U	R.EGLTSIEEVTK.N
645	616.3176	1230.6206	1230.6204	0.17	1	31	0.012	1	U	R.SKEQAELEAAR.Q
663	621.8439	1241.6732	1241.6728	0.32	0	65	4e-006	1	U	R.QVQVALETAQR.S
701	642.3148	1282.6151	1282.6153	-0.19	0	(26)	0.029	1	U	R.QLAEEDLAQQR.A
703	642.8616	1283.7086	1283.7085	0.08	0	42	0.00066	1	U	R.SLVPAAELLESR.V
709	644.3487	1286.6828	1286.6830	-0.14	0	64	5.6e-006	1	U	K.AQVEQELTTLR.L
717	648.8071	1295.5997	1295.5994	0.28	0	57	2.3e-005	1	U	K.GGELVYTDSEAR.D
720	650.3201	1298.6257	1298.6255	0.16	0	53	5.3e-005	1	U	R.QQGLASYDYVR.R
721	650.3853	1298.7561	1298.7558	0.21	0	27	0.015	1	U	K.VTLVQTLEIQR.Q

723 650.8283 1299.6419 1299.6419 0.07 0 44 0.00048 1 U R.QLAEEDLAQQR.A
790 675.7816 1349.5487 1349.5484 0.21 0 46 7.8e-005 1 U R.DSQDAGGFGPEDR.L
856 482.2430 1443.7073 1443.7066 0.49 1 30 0.014 1 U R.LRAETEQGEQQR.Q
880 735.3469 1468.6792 1468.6794 -0.14 0 38 0.0021 1 U R.QQEELLAEENQR.L
890 739.3699 1476.7252 1476.7242 0.65 0 28 0.029 1 U R.SQVMDEATALQLR.E
920 766.4463 1530.8780 1530.8770 0.70 0 26 0.013 1 U K.VLALPEPSPAAPTLR.S
929 778.9175 1555.8204 1555.8205 -0.09 0 34 0.0054 1 U R.LQEAGILSAEELQR.L
939 783.9460 1565.8774 1565.8777 -0.18 0 82 4e-008 1 U R.APVPASELLASGVLSR.A
966 807.4409 1612.8672 1612.8672 -0.02 0 56 2.6e-005 1 U R.LLDAQLSTGGIVDPSK.S
1012 854.9173 1707.8200 1707.8203 -0.15 0 41 0.0014 1 U R.LLDPEDVDVPQPDEK.S
1047 595.3107 1782.9102 1782.9112 -0.55 0 28 0.021 1 U R.AALAHSEEVTASQVAATK.T
1065 904.9543 1807.8940 1807.8952 -0.66 0 67 3.7e-006 1 U K.VQSGSESVIQEYVDLR.T

Proteomics Dataset S2: Related to Figure 3: Proteomics analysis of PCS2 pulldown gel band at approximately 450 kDa. Mascot analysis lists the top scoring peptides from the search. Majority of the results contain common contaminants proteins such as keratin. Plectin was the potential prediction, both showing a high score and sufficient number of queries sufficient to justify investigating further.

Proteomics sequencing data 3:

Mascot Search Results

User : Discoverer_Velos Pro
Search title : 081216gu349-gomika1_Node:2
MS data file : File Name: 081216gu349-gomika1.raw
Database : SwissProt 2016_07 (551705 sequences; 197114987 residues)
Taxonomy : Homo sapiens (human) (20198 sequences)
Timestamp : 13 Aug 2016 at 01:43:59 GMT

Protein hits :
K2C1_HUMAN Keratin, type II cytoskeletal 1 OS=Homo sapiens GN=KRT1 PE=1 SV=6
K1C9_HUMAN Keratin, type I cytoskeletal 9 OS=Homo sapiens GN=KRT9 PE=1 SV=3
PLEC_HUMAN Plectin OS=Homo sapiens GN=PLEC PE=1 SV=3
K1C10_HUMAN Keratin, type I cytoskeletal 10 OS=Homo sapiens GN=KRT10 PE=1 SV=6
K22E_HUMAN Keratin, type II cytoskeletal 2 epidermal OS=Homo sapiens GN=KRT2 PE=1 SV=2
AHNK_HUMAN Neuroblast differentiation-associated protein AHNAK OS=Homo sapiens GN=AHNAK PE=1 SV=2
ALBU_HUMAN Serum albumin OS=Homo sapiens GN=ALB PE=1 SV=2
EPIPL_HUMAN Epiplakin OS=Homo sapiens GN=EPPK1 PE=1 SV=2
CASP_HUMAN Protein CASP OS=Homo sapiens GN=CUX1 PE=1 SV=2
EF1A1_HUMAN Elongation factor 1-alpha 1 OS=Homo sapiens GN=EEF1A1 PE=1 SV=1
LRMP_HUMAN Lymphoid-restricted membrane protein OS=Homo sapiens GN=LRMP PE=1 SV=3
ITA6_HUMAN Integrin alpha-6 OS=Homo sapiens GN=ITGA6 PE=1 SV=5

Mascot Score Histogram
Ions score is -10*Log(P), where P is the probability that the observed match is a random event.
Individual ions scores > 21 indicate identity or extensive homology (p<0.05).
Protein scores are derived from ions scores as a non-probabilistic basis for ran king protein hits.
Score Distribution

Archive Report of Selected Matches

3. PLEC_HUMAN **Mass:** 531466 **Score:** 455 **Matches:** 21(20) **Sequences:** 18(17) **emPAI:** 0.17
Plectin OS=Homo sapiens GN=PLEC PE=1 SV=3

Query	Observed	Mr(expt)	Mr(calc)	ppm	Miss	Score	Expect	Rank	Unique	Peptide
140	459.7560	917.4975	917.4971	0.43	0	58	2.1e-005	1		R.VPVDVAYR.R
208	492.7589	983.5033	983.5036	-0.31	0	59	1.3e-005	1	U	R.VPDVQDGVR.A
250	510.7954	1019.5763	1019.5764	-0.09	0	34	0.0027	1	U	K.LSVYAALQR.Q
319	539.7622	1077.5097	1077.5125	-2.51	0	27	0.018	1	U	R.SMVEEGTGLR.L
364	555.8104	1109.6063	1109.6080	-1.59	0	36	0.0017	1	U	K.AQLEPVASPAK.K
404	586.3343	1170.6541	1170.6496	3.91	0	22	0.069	1	U	R.QVEEEILALK.A
420	593.8262	1185.6379	1185.6353	2.20	1	28	0.018	1	U	K.RQELEAELAK.V
424	595.8458	1189.6771	1189.6707	5.39	0	43	0.00037	1	U	R.LLFNDVQTLK.D
464	621.8439	1241.6733	1241.6728	0.42	0	(31)	0.0094	1	U	R.QVQVALETAQR.S
465	621.8451	1241.6756	1241.6728	2.28	0	33	0.0058	1	U	R.QVQVALETAQR.S
498	642.8641	1283.7137	1283.7085	4.09	0	44	0.00034	1	U	R.SLVPAAELLESR.V
501	643.8470	1285.6795	1285.6779	1.29	0	55	4e-005	1	U	R.WQAVLAQTDVR.Q
508	650.3860	1298.7575	1298.7558	1.35	0	57	1.1e-005	1	U	K.VTLVQTLEIQR.Q
524	661.8371	1321.6596	1321.6514	6.25	0	46	0.00034	1	U	R.SQVEEELFSVR.V
600	731.3731	1460.7315	1460.7293	1.53	0	69	1.6e-006	1	U	R.SQVMDEATALQLR.E

636 769.9319 1537.8492 1537.8464 1.82 0 38 0.0011 1 U R.LLDAQLATGGIVDPR.L
646 778.9189 1555.8233 1555.8205 1.79 0 (26) 0.028 1 U R.LQEAGILSAEELQR.L
647 778.9190 1555.8235 1555.8205 1.88 0 28 0.018 1 U R.LQEAGILSAEELQR.L
663 807.4424 1612.8703 1612.8672 1.94 0 64 4.3e-006 1 U R.LLDAQLSTGGIVDPSK.S
664 807.4456 1612.8767 1612.8672 5.88 0 (34) 0.0038 1 U R.LLDAQLSTGGIVDPSK.S
699 854.9209 1707.8272 1707.8203 4.06 0 26 0.032 1 U R.LLDPEDVDVPQPDEK.S

6. AHNK_HUMAN **Mass:** 628699 **Score:** 119 **Matches:** 7(7) **Sequences:** 7(7) **emPAI:** 0.06
Neuroblast differentiation-associated protein AHNAK OS=Homo sapiens GN=AHNAK PE=1 SV=2

Query	Observed	Mr(expt)	Mr(calc)	ppm	Miss	Score	Expect	Rank	Unique	Peptide
405	586.8433	1171.6721	1171.6635	7.34	1	24	0.033	1	U	K.MKGDVVVSLPK.V
485	634.3311	1266.6477	1266.6456	1.67	0	39	0.0016	1	U	K.AEGPEVDVNLPK.A
565	692.3627	1382.7109	1382.7042	4.88	0	28	0.023	1	U	K.VDINAPDVEVQGK.V
638	514.2855	1539.8347	1539.8330	1.10	1	28	0.014	1	U	K.ISMPDIDLNLKGPK.V
684	834.9235	1667.8324	1667.8254	4.17	0	43	0.0007	1	U	K.VDVEVPDVSLEGPEGK.L
688	841.4195	1680.8244	1680.8207	2.24	0	27	0.024	1	U	K.VDVDVPDVNIEGPDAK.L
694	848.4264	1694.8382	1694.8363	1.14	0	42	0.00089	1	U	K.VDIDVPDVNIEGPDAK.L

Search Parameters
Type of search : MS/MS Ion Search
Enzyme : Trypsin
Variable modifications : Oxidation (M),Gln->pyro-Glu (N-term Q),Trioxidation (C)
Mass values : Monoisotopic
Protein Mass : Unrestricted
Peptide Mass Tolerance : ± 10 ppm
Fragment Mass Tolerance: ± 0.8 Da
Max Missed Cleavages : 2
Instrument type : ESI-TRAP
Number of queries : 992

Proteomics Dataset S3: Related to Figure 3: Proteomics analysis of PCS2 pulldown gel band at approximately 500 kDa, on a second attempt pulldown. Mascot analysis lists the top scoring peptides from the search. Majority of the results contain common contaminants proteins such as keratin. Plectin and AHNK were the potential predictions, both showing a high score and sufficient number of queries. AHNK was not present in other attempts, however, implying it to be a false positive.

Proteomics sequencing data 4:

Mascot Search Results

User : Discoverer_Velos Pro
Search title : 081216gu349-gomika2_Node:2
MS data file : File Name: 081216gu349-gomika2.raw
Database : SwissProt 2016_07 (551705 sequences; 197114987 residues)
Taxonomy : Homo sapiens (human) (20198 sequences)
Timestamp : 13 Aug 2016 at 21:33:13 GMT

Protein hits :
K2C1_HUMAN Keratin, type II cytoskeletal 1 OS=Homo sapiens GN=KRT1 PE=1 SV=6
K1C10_HUMAN Keratin, type I cytoskeletal 10 OS=Homo sapiens GN=KRT10 PE=1 SV=6
K1C9_HUMAN Keratin, type I cytoskeletal 9 OS=Homo sapiens GN=KRT9 PE=1 SV=3
K22E_HUMAN Keratin, type II cytoskeletal 2 epidermal OS=Homo sapiens GN=KRT2 PE=1 SV=2
K2C6B_HUMAN Keratin, type II cytoskeletal 6B OS=Homo sapiens GN=KRT6B PE=1 SV=5
K1C14_HUMAN Keratin, type I cytoskeletal 14 OS=Homo sapiens GN=KRT14 PE=1 SV=4
MYH9_HUMAN Myosin-9 OS=Homo sapiens GN=MYH9 PE=1 SV=4
K2C6A_HUMAN Keratin, type II cytoskeletal 6A OS=Homo sapiens GN=KRT6A PE=1 SV=3
K1C16_HUMAN Keratin, type I cytoskeletal 16 OS=Homo sapiens GN=KRT16 PE=1 SV=4
K2C5_HUMAN Keratin, type II cytoskeletal 5 OS=Homo sapiens GN=KRT5 PE=1 SV=3
ALBU_HUMAN Serum albumin OS=Homo sapiens GN=ALB PE=1 SV=2

Mascot Score Histogram
Ions score is -10*Log(P), where P is the probability that the observed match is a random event.
Individual ions scores > 21 indicate identity or extensive homology (p<0.05).
Protein scores are derived from ions scores as a non-probabilistic basis for ran king protein hits.
Score Distribution

Archive Report of Selected Matches

7. MYH9_HUMAN **Mass:** 226392 **Score:** 372 **Matches:** 19(17) **Sequences:** 16(14) **emPAI:** 0.36
Myosin-9 OS=Homo sapiens GN=MYH9 PE=1 SV=4
Query Observed Mr(expt) Mr(calc) ppm Miss Score Expect Rank Unique Peptide
51 **423.7010 845.3874 845.3879 -0.55 0 27 0.01 1 U K.DQGELER.Q**
149 **462.7502 923.4858 923.4865 -0.76 0 (35) 0.0031 1 U R.VVFQEFR.Q**
150 **462.7502 923.4859 923.4865 -0.63 0 44 0.00041 1 U R.VVFQEFR.Q**
440 **597.3107 1192.6069 1192.6088 -1.56 0 49 0.00017 1 U K.ALELDSNLYR.I**
441 **597.3118 1192.6090 1192.6088 0.19 0 (48) 0.00024 1 U K.ALELDSNLYR.I**
455 **603.3238 1204.6331 1204.6339 -0.65 0 31 0.011 1 U K.TDLLLEPYNK.Y**
456 **603.3254 1204.6362 1204.6339 1.89 0 (29) 0.015 1 U K.TDLLLEPYNK.Y**
542 **653.3391 1304.6635 1304.6612 1.80 0 36 0.0031 1 U K.EQADFAIEALAK.A**
571 **666.3098 1330.6051 1330.6000 3.78 0 27 0.016 1 U R.QLEEAEEEAQR.A**
698 **765.8865 1529.7584 1529.7573 0.73 0 50 0.00014 1 U K.IAQLEEQLDNETK.E**
709 **783.3427 1564.6709 1564.6675 2.17 0 56 1.2e-005 1 U R.ELEDATETADAMNR.E**
713 **524.6248 1570.8525 1570.8468 3.63 0 31 0.008 1 U K.VSHLLGINVTDFTR.G**
717 **796.3554 1590.6961 1590.6944 1.11 0 36 0.001 1 U R.NTDQASMPDNTAAQK.V**
751 **863.9810 1725.9475 1725.9413 3.57 0 79 1e-007 1 R.QLLQANPILEAFGNAK.T**
752 **864.4334 1726.8521 1726.8487 1.98 0 23 0.065 1 U K.NLPIYSEEIVEMYK.G**
793 **935.4898 1868.9651 1868.9592 3.16 0 36 0.0034 1 U K.ANLQIDQINTDLNLER.S**
811 **973.5100 1945.0053 1945.0004 2.55 0 21 0.099 1 U K.LQVELDNVTGLLSQSDSK.S**
813 **650.6752 1949.0037 1948.9854 9.37 0 47 0.00023 1 U R.LQQELDDLLVDLDHQR.Q**

```
960 831.7291 2492.1654 2492.1667 -0.52 0 31 0.0086 1 U K.DFSALESQLQDTQELLQEENR.Q

Search Parameters
Type of search : MS/MS Ion Search
Enzyme : Trypsin
Variable modifications : Oxidation (M),Gln->pyro-Glu (N-term Q),Trioxidation (C)
Mass values : Monoisotopic
Protein Mass : Unrestricted
Peptide Mass Tolerance : ± 10 ppm
Fragment Mass Tolerance: ± 0.8 Da
Max Missed Cleavages : 2
Instrument type : ESI-TRAP
Number of queries : 1019
```

Proteomics Dataset S4: Related to Figure 3: Proteomics analysis of PCS2 pulldown gel band at approximately 250 kDa, on the same pulldown as S3. Mascot analysis lists the top scoring peptides from the search. Majority of the results contain common contaminants proteins such as keratin. MYH9 was the potential prediction, showing a high score and sufficient number of queries. MYH9 was not present in other attempts, however, implying it to be a false positive.

Proteomics sequencing data 5:

Mascot Search Results

User : Discoverer_Velos Pro
Search title : 080216GU289-PCS2D1_Node:2
MS data file : File Name: 080216GU289-PCS2D1.raw
Database : SwissProt 2016_07 (551705 sequences; 197114987 residues)
Taxonomy : Homo sapiens (human) (20198 sequences)
Timestamp : 3 Aug 2016 at 05:23:56 GMT
Enzyme : Trypsin
Variable modifications : Oxidation (M),Gln->pyro-Glu (N-term Q),Trioxidation (C)
Mass values : Monoisotopic
Protein Mass : Unrestricted
Peptide Mass Tolerance : ± 10 ppm
Fragment Mass Tolerance: ± 0.8 Da
Max Missed Cleavages : 2
Instrument type : ESI-TRAP
Number of queries : 700

Protein hits :
K2C1_HUMAN Keratin, type II cytoskeletal 1 OS=Homo sapiens GN=KRT1 PE=1 SV=6
K22E_HUMAN Keratin, type II cytoskeletal 2 epidermal OS=Homo sapiens GN=KRT2 PE=1 SV=2
K1C10_HUMAN Keratin, type I cytoskeletal 10 OS=Homo sapiens GN=KRT10 PE=1 SV=6
K1C9_HUMAN Keratin, type I cytoskeletal 9 OS=Homo sapiens GN=KRT9 PE=1 SV=3
K1C14_HUMAN Keratin, type I cytoskeletal 14 OS=Homo sapiens GN=KRT14 PE=1 SV=4
K2C5_HUMAN Keratin, type II cytoskeletal 5 OS=Homo sapiens GN=KRT5 PE=1 SV=3
DCD_HUMAN Dermcidin OS=Homo sapiens GN=DCD PE=1 SV=2
PLEC_HUMAN Plectin OS=Homo sapiens GN=PLEC PE=1 SV=3
ALBU_HUMAN Serum albumin OS=Homo sapiens GN=ALB PE=1 SV=2
DJC15_HUMAN DnaJ homolog subfamily C member 15 OS=Homo sapiens GN=DNAJC15 PE=1 SV=2
LRMP_HUMAN Lymphoid-restricted membrane protein OS=Homo sapiens GN=LRMP PE=1 SV=3
SHRM3_HUMAN Protein Shroom3 OS=Homo sapiens GN=SHROOM3 PE=1 SV=2

SwissProt Decoy False discovery rate
Peptide matches above identity threshold 108 23 21.30 %
Peptide matches above homology or identity threshold 108 28 25.93 %

Select Summary Report
Select Summary (protein hits)
Significance threshold p< Max. number of hits Show Percolator scores
Standard scoring MudPIT scoring Ions score or expect cut-off Show sub-sets
Show pop-ups Suppress pop-ups Require bold red

Preferred taxonomy All entries

All queries Unassigned Below homology threshold Below identity threshold
8. PLEC_HUMAN **Mass:** 531466 **Score:** 44 **Matches:** 1(1) **Sequences:** 1(1) **emPAI:** 0.01
Plectin OS=Homo sapiens GN=PLEC PE=1 SV=3
Query Observed Mr(expt) Mr(calc) ppm Miss Score Expect Rank Unique Peptide
261 508.2797 1014.5448 1014.5458 -0.93 0 44 0.00044 1 U R.LSVAAQEAAR.L

Proteomics Dataset S5: Related to Figure 3: Proteomics analysis of PCS2 pulldown gel at approximately 250 kDa on a third attempt pulldown. Mascot analysis lists the top scoring peptides from the search. Majority of the results contain common contaminants proteins such as keratin, but Plectin is present in these results as well.

References:

Matharage, J.M., Minna, J.D., Brekken, R.A., and Udugamasooriya, D.G. (2015). Unbiased Selection of Peptide–Peptoid Hybrids Specific for Lung Cancer Compared to Normal Lung Epithelial Cells. ACS Chem Biol *10*, 2891-2899.

Additional Experimental Details and Procedures:

Cell lines: Lung Cancer cell lines included the epithelial lung cell line HBEC3-KT (female), and the following NSCLC lines: NCI-H2009 (female), HCC-4017 (female), NCI-H460 (male), NCI-H1975 (female), NCI-2122 (female), NCI-H2017 (female), NCI-H1993 (female), NCI-H1299 (male), NCI-H1155 (male), NCI-H1395 (female), NCI-H358 (male), and NCI-H693 (female). All lung cancer cell lines and HBEC3KT normal cells were obtained from the cell collection of Dr. John Minna's research group at the UT Southwestern Medical Center, and confirmed by DNA fingerprinting at that facility. Lung cancer cell lines were grown in RPMI supplemented with 10% FBS under 37°C and 5% CO_2. Normal lung cell line HBEC3-KT were grown in keratinocyte serum-free media supplemented with human recombinant epidermal growth factor and bovine pituitary extract (KSFM complete media, Thermo Fisher), under 37°C and 5% CO_2.

Human adipose derived Mesenchymal Stem Cells were obtained from ATCC (PCS-500-011), and were grown in Mesenchymal Stem Cell Basal Medium (ATCC PCS-500-300) with added supplement Mesenchymal Stem Cell Growth Kit (ATCC PCS-500-40) under 37°C and 5% CO_2.

Gene expression analysis: Each time when performing RT-qPCR, cells were lysed in 100 µL iScript RT-qPCR Sample Prep Reagent (Bio-Rad), vortexed for 30 seconds and then centrifuged. Twelve µL of the lysate was added to iScript cDNA synthesis kit (Bio-Rad) to a total volume of 20 µL and run for 1 hour at 42°C. 2 µL of each cDNA was added to a mixture containing: 17.5 µL Fast Start Essential DNA Probes Master, 13.75 µL water, 1.75 µL TaqMan *GAPDH* (VIC) probe (Thermo Fisher), and 1.75 µL of either Hs00167476_m1 TaqMan *ALDH1A3* (FAM), Hs01053049_s1 TaqMan *SOX2* (FAM), Hs01075864_m1 TaqMan *CD44* (FAM), or Hs00356986_g1 TaqMan *PLEC* (FAM) probe (Thermo Fisher). Each sample was then mixed, pipetted in triplicate at a volume of 10 µL per well, and run on a Lightcycler 96 (Roche) under the program: 95°C for 10 minutes and 40 cycles of 95°C for 10 sec and 60°C for 30 seconds. Relative expression of each sample was calculated using the ddCT method, with *GAPDH* expression as the normalization control.

Plectin siRNA Knockdown: Cells were plated on a 6-well plate and grown to 80% confluence. For each well, 9 µL of Lipofectamine RNAiMAX (Thermo Fisher) was mixed with 3 µL of 10 µM siRNA, either silencer select s10644 plectin or silencer select negative control 2 (Thermo Fisher), in serum-free RPMI and incubated for 5 minutes. The cells were then grown for 48 hours, and then used for clonogenicity experiments following the above protocol.

Mobility "Scratch Assay": Cells were plated in 24-well plates, and incubated under normal conditions until 80% confluent, at which point each well was transfected with siRNA using a scaled down (1/3) variation of the protocol described above. After 36 hours of incubation the wells were scratched using a 10uL tip, washed and media was replenished and incubated for an additional 24 hours. Each well was

then fixed with Formalin for 30 minutes, and stained with Crystal Violet for 5 minutes, before being washed with MilliQ-filtered dH_2O. The "scratch" width remaining was then photographed by bright field view on our BX-51 fluorescence microscope (Olympus) and the average area remaining was calculated. This experimental protocol was also performed with the following incubations with siRNA: 48 hours pre-scratch and 24 hours post-scratch, and 96 hours with no scratch.

Cyanogen Bromide (CNBr) cleavage of beads (for library and later bulk synthesis on tentagel beads): Small amounts of beads were removed from reaction vessels before storage and washed with DCM (2ml x 3 times). Cleavage mixture (or cocktail) was prepared by adding 30µl of CNBr (2M solution in Acetonitrile) to 1ml solution of Acetonitrile and water (7:3). 50µl of the cleavage mixture was then added to the beads and kept on the shaker overnight. CNBr solution was allowed to evaporate and 1:1 mixture of acetonitrile and water was added to the beads and resulting solution was used to confirm mass of the compound.

ALDEFluor staining and CSC sorting: The Aldefluor assay (Stem Cell Technologies) was used to profile, sort and stain cells based on ALDH activity. H358 lung cancer cells were incubated in ALDEFluor assay buffer containing the ALDH protein substrate BODIPY-aminoacetaldehyde (BAAA) for 45 minutes at 37° at a concentration of 106 cells per mL. ALDH-specific inhibitor diethylaminobezaldehyde (DEAB) addition during incubation was used as a negative control sample and for gating determination. Cells were sorted by BD Aria (BD Biosciences) cell sorters at core facilities in University of Texas Southwestern Medical Center and the University of Texas MD Anderson Cancer Center. The isolated ALDH+ fraction was isolated to be the cells with higher fluorescence due to ALDEFluor than the highest fluorescence seen in the ALDH inhibitor(DEAB)-treated sample, to a maximum of 10% of the original cell population, and the ALDH- fraction was isolated as 10% of cells with the lowest fluorescence.

QDot cell labeling procedure for OTC assay: Cells were counted and distributed in three 1.5ml microcentrifuge tubes (total of six tubes for both cell groups) with 1 x 106 cells in 1 ml of media per each tube. To prepare 10nM labeling solution (typical working concentration is 2-15nM), pre-mix 1µl each of Qtracker reagent (Invitrogen) A and B in a 1.5ml microcentrifuged tubes (prepared three tubes for each color) and incubated for 5 minutes at room temperature. 0.2ml of respective medium was added to each tube and vortexed for 30 seconds. 1 X 10^6 cells were added to each tube (three ALDH+ and three ALDH-) containing the labeling solution and incubated at 37°C for 60 minutes.

On-bead TentaGel cell binding assay for qualitative binding confirmation of $ALDH^+$ CSC cells to PCS2 compound: This assay was performed at 3 days and 14 after ALDH sorting. 200 µl of TentaGel beads containing PCS2 compound were transferred into each of two 1.5 mL microcentrifuge tubes. The beads were washed 2 times in RPMI medium with 5% FBS and equilibrated in same medium containing 1% BSA for 1 hour. Previously sorted H358 $ALDH^+$ and $ALDH^-$ cells were removed from culture plates, counted and 0.5 x10^6 cells from each cell types were labeled with Qdot. Half the amount of each H358 $ALDH^+$ and $ALDH^-$ cells were continuously grown (with appropriate splitting) for two weeks to be used in day 14 experiment. After staining, cells were suspended in 1 mL of RPMI medium containing 5% FBS, 1% BSA and pipetted several times to break cell clumps. Red and green cells were separately added to two tubes. Cell density for each cell type was kept as 0.5x10^6 cells/mL in each tube. The beads were incubated at room temperature with gentle shaking for 30 minutes. Finally, the beads were gently washed and visualized under the fluorescent microscope equipped with the DAPI filter.

Cell Surface Fraction western blotting: The cell surface and cytosolic protein fractions were isolated using the Pierce Cell Surface Protein Isolation Kit (Thermo Fisher), following the manufacturer's protocol. In brief, four T75 cm2 flasks of 90-95% confluent cells were washed with ice cold PBS twice. Each vial of sulfo-NHS-biotin compound (12 mg/vial) was dissolved in ice cold PBS (48 mL), and 10 mL of this solution was then added the cells. The cells were incubated for 30 min at 4oC on a rocking platform. This was followed by the addition of 500 µL/flask of quenching solution. The cells were scrapped off the flasks and centrifuged at 500 x g for 3 min. The pellet was washed twice with TBS, before the addition of 500

µL of lysis buffer. The lysate was then sonicated on ice using 1 second bursts and vortexed every 5 min during a 30 min period. The lysate was centrifuged at 10,000 x g for 2 min at 4oC, the clarified supernatant was collected and the pellet was discarded. A column consisting of NeutrAvidin agarose slurry (500 µL) was prepared using SnapCap spin column (Thermo Scientific, Rockford, IL). The column was washed three times using wash buffer before a 60 min end-over-end mixing incubation at room temperature with the clarified cell lysate. The column was centrifuged at 1000 x g and the flow through containing the cytosolic unbiotinylated sample was saved. The column was washed six times with wash buffer. Cell surface biotinylated samples were eluted upon a 60 min end-over-end mixing incubation at room temperature with 400 µL of PBS containing 62.5 mM Tris-HCl and 50 nM dithiothreitol (DTT). The proteins were then collected by a 2 min centrifugation at 1000 x g. The samples were concentrated (10 X) by lyophilization. Blotting and visualizing was performed using the ScanLater Western blot kit (Molecular Devices), as described above, using the anti-plectin antibody and anti-beta-Actin antibody (10004-978, Biovision, San Francisco, CA).
